# The conserved actinobacterial transcriptional regulator FtsR controls expression of *ftsZ* and further target genes and influences growth and cell division in *Corynebacterium glutamicum*

**DOI:** 10.1186/s12866-019-1553-0

**Published:** 2019-08-05

**Authors:** Kim Julia Kraxner, Tino Polen, Meike Baumgart, Michael Bott

**Affiliations:** IBG-1: Biotechnology, Institute for Bio- und Geosciences, Forschungszentrum Jülich, 52425 Jülich, Germany

**Keywords:** DNA affinity chromatography, Transcriptional regulation, ChAP-Seq, *Corynebacterium diphtheriae*, *Mycobacterium tuberculosis*, Rv1828

## Abstract

**Background:**

Key mechanisms of cell division and its regulation are well understood in model bacteria such as *Escherichia coli* and *Bacillus subtilis.* In contrast, current knowledge on the regulation of cell division in *Actinobacteria* is rather limited. FtsZ is one of the key players in this process, but nothing is known about its transcriptional regulation in *Corynebacterium glutamicum,* a model organism of the *Corynebacteriales*.

**Results:**

In this study, we used DNA affinity chromatography to search for transcriptional regulators of *ftsZ* in *C. glutamicum* and identified the Cg1631 protein as candidate, which was named FtsR. Both deletion and overexpression of *ftsR* caused growth defects and an altered cell morphology. Plasmid-based expression of native *ftsR* or of homologs of the pathogenic relatives *Corynebacterium diphtheriae* and *Mycobacterium tuberculosis* in the Δ*ftsR* mutant could at least partially reverse the mutant phenotype. Absence of *ftsR* caused decreased expression of *ftsZ*, in line with an activator function of FtsR. In vivo crosslinking followed by affinity purification of FtsR and next generation sequencing of the enriched DNA fragments confirmed the *ftsZ* promoter as in vivo binding site of FtsR and revealed additional potential target genes and a DNA-binding motif. Analysis of strains expressing *ftsZ* under control of the gluconate-inducible *gntK* promoter revealed that the phenotype of the Δ*ftsR* mutant is not solely caused by reduced *ftsZ* expression, but involves further targets.

**Conclusions:**

In this study, we identified and characterized FtsR as the first transcriptional regulator of FtsZ described for *C. glutamicum*. Both the absence and the overproduction of FtsR had severe effects on growth and cell morphology, underlining the importance of this regulatory protein. FtsR and its DNA-binding site in the promoter region of *ftsZ* are highly conserved in *Actinobacteria*, which suggests that this regulatory mechanism is also relevant for the control of cell division in related *Actinobacteria*.

**Electronic supplementary material:**

The online version of this article (10.1186/s12866-019-1553-0) contains supplementary material, which is available to authorized users.

## Background

Bacterial reproduction is usually characterized by cellular growth followed by binary fission of a mother cell into two daughter cells. During this process, the bacteria need to coordinate several distinct processes such as DNA replication, biogenesis of the new cell wall, and the division process itself. Although the overall process is similar, several different concepts have evolved in distinct bacterial groups. *Escherichia coli* and *Bacillus subtilis* represent two of the best studied species in this respect [1, 2]. *Corynebacterium glutamicum* is a non-pathogenic, facultative anaerobic, Gram-positive soil bacterium frequently used as model organism for the order *Corynebacteriales*, which includes important pathogens such as *Corynebacterium diphtheriae* and *Mycobacterium tuberculosis* causing the fatal infectious diseases diphtheria and tuberculosis [3–6]. *C. glutamicum* strains are used in industrial biotechnology for the production of l-amino acids and other metabolites, as well as for proteins [7–11].

One of the key players of cell division is the highly conserved and essential FtsZ protein, which is present in most bacteria. It is the first protein that moves to the future division site, polymerizes into a ring structure, and serves as scaffold for the assembly of further cell division proteins [12]. The resulting protein complex is called the divisome and drives the constriction during cell division [13]. Interestingly, *Actinobacteria* such as *C. glutamicum* lack homologs of several important cell division-related genes described for *E. coli* or *B. subtilis*. One of these is MreB, an actin homolog essential for lateral cell wall elongation in *E. coli, Caulobacter crescentus*, and *B. subtilis* [14–16]. In contrast to these species, *C. glutamicum* inserts new cell wall material at the poles, representing an apical elongation mechanism [17]. Another protein absent in *C. glutamicum* is FtsN*,* an essential membrane protein of *E. coli* with a potential role in coordination of cell division [1, 18]. However, most strikingly is the complete absence of homologs of many known spatial and temporal, positive and negative regulators of cell division such as *ftsA, ezrA*, *noc*/*slmA*, *zapA*, and *minCDE* [18, 19]. This implies that cell division in the *Corynebacteriales* and probably other *Actinobacteria* is regulated through proteins and mechanisms that differ significantly from previously described ones.

To date, we have only a limited understanding of the regulation of cytokinesis in *Corynebacteriales* [18]. Accurate expression of *ftsZ* is critical for normal growth of *C. glutamicum* and *M. tuberculosis* and small variations lead to severe morphological changes and affect cell viability [19–21]. Thus, the FtsZ level has to be tightly regulated, which is supported by different studies of the *ftsZ* promoter region showing a very complex transcription of *ftsZ* in various actinobacteria [19, 22–24]. Current knowledge on transcriptional regulation of *ftsZ* expression in *C. glutamicum* is sparse. A complex of the DNA-binding proteins WhiA (Cg1792) and WhcD (Cg0850) was reported to bind to the *ftsZ* promoter region [25], however, the effect of this interaction on *ftsZ* expression remains to be elucidated. We previously showed that the FtsZ protein can be phosphorylated in vitro by the serine/threonine protein kinases PknA, PknB, and PknL and is an in vivo substrate of the phospho-Ser/Thr protein phosphatase Ppp [26], suggesting that the properties and activities of FtsZ can also be influenced by posttranslational regulation [18].

In this study, we identified and characterized the transcriptional regulator Cg1631 of *C. glutamicum* as an activator of *ftsZ* expression. Therefore, Cg1631 was renamed FtsR, standing for *ftsZ* regulator. We show that FtsR is critical for normal growth and cell morphology of *C. glutamicum*, suggesting an important role of this protein in the regulation of cell division.

## Results

### Search for transcriptional regulators of *ftsZ*

**Fig. 1:**
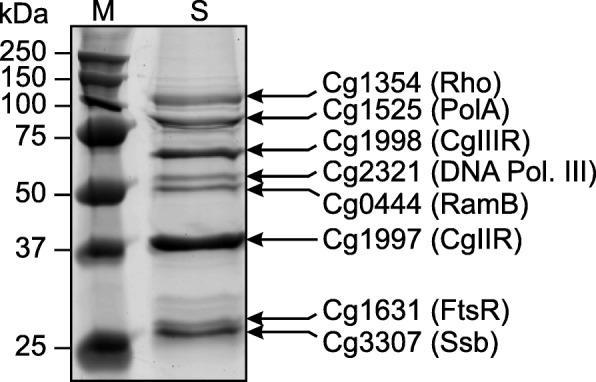
DNA affinity chromatography with the *ftsZ* promoter region. Crude cell extract of *C. glutamicum* ATCC13032 cultivated in glucose minimal medium to the mid-exponential growth phase was incubated with an immobilized 417-bp DNA fragment covering the *ftsZ* promoter region and strongly binding proteins were eluted with a high-salt buffer. Proteins enriched with the *ftsZ* promoter were separated by SDS-PAGE, stained with colloidal Coomassie, and identified by peptide-mass fingerprinting using MALDI-ToF MS analysis (Additional file 1: Table S1). The band with an apparent mass of about 27 kDa was identified as FtsR. M, marker; S, sample

To investigate transcriptional control of *ftsZ*, we searched for potential regulators by DNA affinity chromatography using a 417 bp DNA fragment covering the 3′-end of the *ftsQ* coding region (36 bp), the intergenic region between *ftsQ* and *ftsZ*, and the first 96 bp of the *ftsZ* coding region. The immobilized DNA fragment was incubated with crude protein extract of *C. glutamicum* wild type cells. The proteins eluting after several washing steps were separated by SDS-PAGE. Subsequent MALDI-ToF-MS analysis resulted in the identification of eight proteins (Fig. 1, Additional file 1: Table S1). Six of them represent proteins that are often found in DNA affinity purifications with *C. glutamicum* cell extracts independent of the promoter region used. These are the single-stranded DNA-binding protein Ssb (Cg3307), the restriction endonucleases CglIR (Cg1997) and CglIIR (Cg1998), the ε-subunit of DNA polymerase III (Cg2321), DNA polymerase I (Cg1525), and the transcription termination factor Rho (Cg1354). Most interesting were the remaining two proteins, both of which are annotated as transcriptional regulators, Cg0444 (RamB) and Cg1631. The identity of these two proteins was further verified by MS/MS analysis of selected peptides (Additional file 1: Table S1). The regulator of acetate metabolism RamB (Cg0444) has already been studied quite extensively and represses genes involved in acetate metabolism and alleviates glucose and sucrose uptake [27, 28]. A function in *ftsZ* regulation has not been described for RamB and a well-conserved RamB-binding site (AA/GAACTTTGCAAA [28]) is absent from the *ftsZ* promoter region. In contrast to RamB, the function of the second transcriptional regulator enriched with the *ftsZ* promoter fragment, Cg1631, was completely unknown and Cg1631 was therefore the focus of this study. Due to its role in *ftsZ* regulation, the protein was named FtsR.

### FtsR, a MerR-type transcriptional regulator conserved in *Actinobacteria*

**Fig. 2:**
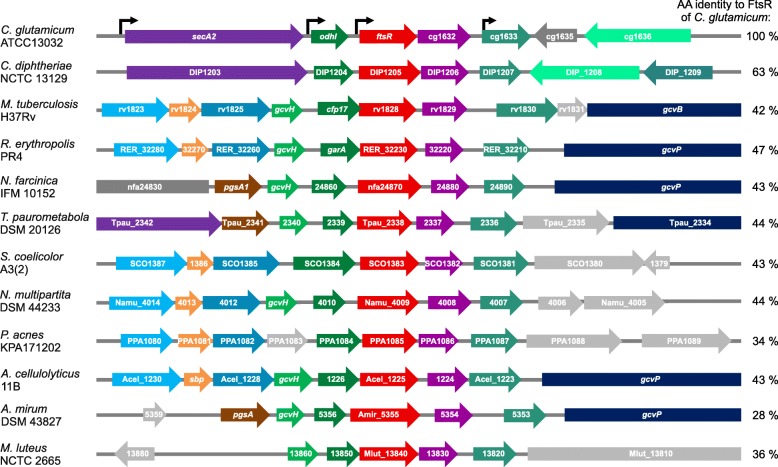
Phylogenetic conservation of *ftsR* (cg1631) and adjacent genes. *ftsR* and its homologs in different actinobacterial species of various genera are shown in red. Neighboring homologous genes are colored alike, whereas grey arrows indicate genes that are not conserved in the gene cluster analyzed. The black arrows indicate transcriptional start sites determined by RNA-Seq for *C. glutamicum* [29]. Note that the four-gene-cluster comprising *ftsR*, its upstream gene *odhI*/*garA*, and the two downstream genes encoding a bifunctional nuclease and a MerR-type transcriptional regulator is strongly conserved. The figure was prepared based on data of MicrobesOnline [30] and ERGO [31]. The amino acid sequence identity of the *C. glutamicum* FtsR homologs is given on the right and was derived from NCBI BLAST [32]. For lack of space, some locus tag prefixes were omitted in short genes. Gene lengths are approximately to scale. The full species names are as following: *Corynebacterium glutamicum*, *Corynebacterium diphtheriae*, *Mycobacterium tuberculosis*, *Rhodococcus erythropolis*, *Nocardia farcinica*, *Tsukamurella paurometabola*, *Streptomyces coelicolor*, *Nakamurella multipartita*, *Propionibacterium acnes*, *Acidothermus cellulolyticus*, *Actinosynnema mirum*, *Micrococcus luteus*

The gene *ftsR* (cg1631) of *C. glutamicum* ATCC13032 encodes a protein of 252 amino acids (calculated mass 27.23 kDa), which was annotated as a transcriptional regulator of the MerR family due to the sequence similarity of the N-terminal region of FtsR to other members of the MerR family. The *ftsR* gene and its immediate genomic vicinity including the upstream gene *odhI* (cg1630) and the two downstream genes (cg1632 and cg1633) were found to be highly conserved within the *Actinobacteria* (Fig. 2). The *odhI* gene encodes the 2-oxoglutarate dehydrogenase inhibitor protein OdhI, which acts as a phosphorylation-dependent switch controlling the activity of the 2-oxoglutarate dehydrogenase complex [33–35]. The homologous protein of mycobacteria is GarA [36]. The gene cg1632 encodes a putative bifunctional nuclease having both DNase and RNase activity (PFAM PF02577), and cg1633 encodes another yet uncharacterized transcriptional regulator of the MerR family. Previous RNAseq analysis indicated that *ftsR* is co-transcribed with these neighboring genes in various combinations: cg1629-cg1633 was annotated as primary operon and cg1630-cg1633 and cg1631-cg1633 as sub-operons [29] (Fig. 2). The genomic region upstream of *odhI* (cg1630) and downstream of cg1633 varies in different *Actinobacteria*.

An amino acid sequence alignment of FtsR homologs of different actinobacterial species revealed that especially the N-terminal part is highly conserved (Additional file 1:Figure S1). Two features of the alignment are conspicuous, which are the variable length of the N-termini upstream of the highly conserved region starting with the sequence MSIG and the variable length of the region linking the N-terminal DNA-binding domain and the C-terminal regulatory domain.

### Deletion and overexpression of *ftsR* affects growth behavior and cell shape

**Fig. 3:**
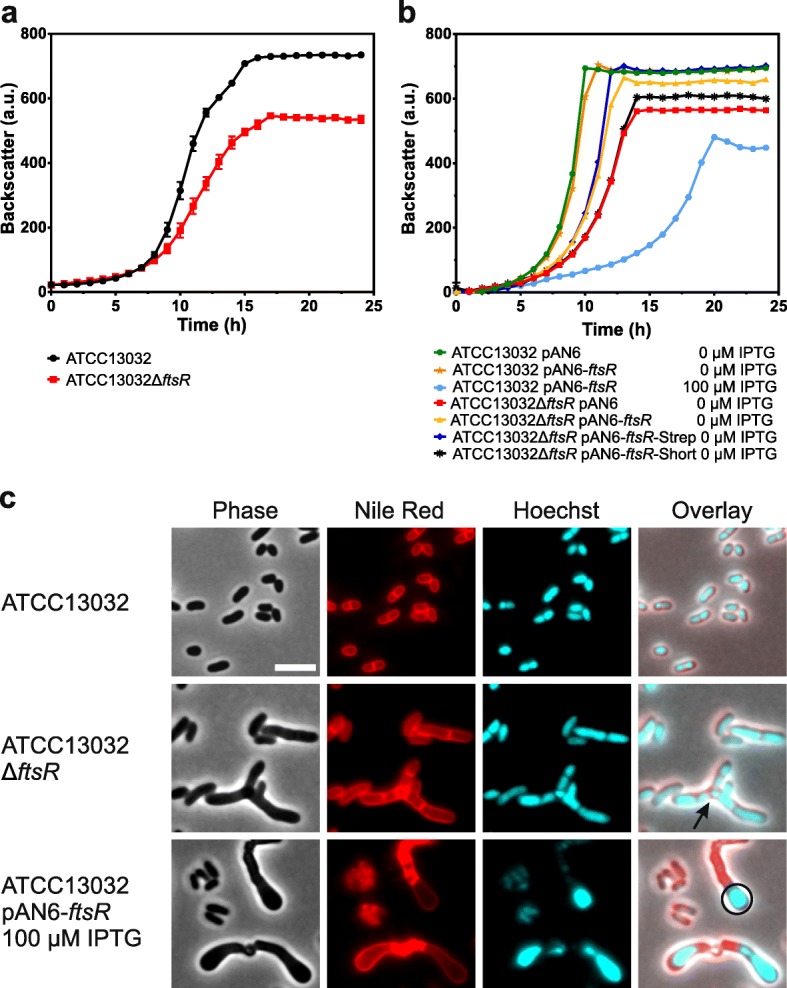
Growth behavior and cell morphology of *C. glutamicum* Δ*ftsR* and *C. glutamicum* overexpressing *ftsR*. **a** Growth of the *ftsR* deletion mutant in comparison to the wild type. Mean values and standard deviation of three biological replicates are shown. **b** Growth of an *ftsR* overexpressing strain and complementation of the Δ*ftsR* mutant with several FtsR variants including an N-terminally shortened protein and a protein with a C-terminal StrepTag-II. Average values from two biological replicates are shown. For the growth experiments shown in **a** and **b**, the strains were pre-cultivated first in BHI medium and then in CGXII medium with 2% (w/v) glucose, followed by the main cultivation in the same medium. For **b**, all media were supplemented with kanamycin (25 μg/mL) and IPTG as indicated. **c** Microscopic pictures of cells in the stationary phase. To visualize membranes and DNA, cells were stained with Nile Red and Hoechst 33342, respectively. The arrow points towards a branched cell and the circle indicates a high DNA concentration at the cell pole. The scale bar represents 5 μm

To gain insights into the function of FtsR in *C. glutamicum*, an in-frame *ftsR* deletion mutant as well as an *ftsR*-overexpressing strain were constructed (Additional file 1: Tables S2 and S3) and analyzed with respect to growth behavior and cell morphology. Remarkably, the *ftsR* deletion strain grew significantly slower and to a lower final cell density (backscatter) compared to the wild type in CGXII minimal medium with glucose as carbon source (Fig. 3a). The consequences of *ftsR* overexpression in the wild-type strain ATCC13032 were tested using plasmid pAN6-*ftsR*, in which *ftsR* expression is controlled by the *tac* promoter. The promoter is known to be leaky in *C. glutamicum* and allows basal expression of the target gene also in the absence of the inducer IPTG [37]. Without IPTG and with IPTG concentrations up to 10 μM, growth of ATCC13032 pAN6-*ftsR* was comparable to the reference strain ATCC13032 pAN6. However, when 100 μM IPTG was added, growth of ATCC13032 pAN6-*ftsR* was strongly inhibited (Fig. 3b). Thus, both the absence and the overexpression of *ftsR* had negative consequences. We searched our in-house microarray database with more than 1700 experiments for conditions with altered *ftsR* mRNA levels, but found no evidence for transcriptional regulation of *ftsR*. Together, these results suggest that *ftsR* expression in the cell may be constitutive.

Phase-contrast and fluorescence microscopy with staining of DNA and membranes revealed strong morphological differences to wild-type cells both for the Δ*ftsR* mutant and for the *ftsR* overexpressing strain (Fig. 3c, Additional file 1: Figure S2). These differences were apparent both in the exponential and stationary growth phases (Additional file 1: Figure S3). While the phenotype of the Δ*ftsR* mutant did not change much along the cultivation, the phenotype of the overexpression strain became more severe over time (Additional file 1: Figure S3). This could be due to an accumulation of FtsR in the cells resulting from the overexpression. The Δ*ftsR* cells were elongated and some of them were branched (Fig. 3c, black arrow). Several cells had multiple septa at different positions in the cell. Overexpression of *ftsR* led to enlarged cells with DNA accumulated at the poles (Fig. 3c, black circle). These cells also contained additional septa, which were mostly located in the middle of the cells. However, only a fraction of the cells was enlarged, whereas the others remained small and inconspicuous. Please note that the small cells appear DNA-free in some pictures because the brightness was adjusted to the cells with the high DNA concentration. All small cells of the FtsR overexpression strain seem to contain similar DNA amounts like the wild type. The reason for this population heterogeneity is unknown at present. It could result from different FtsR levels in the cells due to heterogeneous induction of pAN6-based *ftsR* expression, as previously reported for *eyfp* expression by the pAN6 parent plasmid pEKEx2 [38]. The observed morphological changes caused by *ftsR* deletion and overexpression, in particular the presence of multiple septa within a single cell that did not lead to a separation of the cells, hint toward a function of FtsR in cell division and to a potential role in the regulation of *ftsZ* expression. To further evaluate the changes in cell size or rather cell volume, we analyzed the strains using a Coulter counter in the volumetric measurement mode. Additional file 1:Figure S4 shows the size distribution at three different time points during cultivation (3 h, 6 h, 24 h). For the Δ*ftsR* strain in comparison to the wild type, the whole population was shifted to larger cell sizes at all time points and formed a single broad peak. The population of the *ftsR* overexpressing strain formed two peaks of different sizes at 24 h, differing weakly or strongly in size from wild type carrying pAN6. As mentioned above, this could be due to heterogeneous expression from plasmid pAN6-*ftsR*. Overall, these measurements confirmed that the *ftsR* deletion strain comprises a homogeneous population of cells with increased size in different growth phases. In contrast, the strain overexpressing *ftsR* showed a homogeneous population of enlarged cells in the exponential growth phase, and interestingly two populations with cells of different sizes in the stationary phase, which matches the. These results coincide with the cells size distribution observed by microscopy.

In order to corroborate the results described above, the *ftsR* gene was also deleted and overexpressed in another *C. glutamicum* strain termed MB001, which differs from ATCC13032 by the deletion of the three prophage regions CGP1 (13.5 kb), CGP2 (3.9 kb), and CGP3 (187.3 kb) [39]. We previously reported that the MB001 strain behaved like its parent wild type in the majority of conditions tested, but showed improved growth and fitness under SOS response-inducing conditions that trigger CGP3 induction in the wild type. Thus, MB001 is a useful strain background for the analysis of deletions causing stressed cells, as secondary effects due to prophage induction are excluded. Both growth behavior (Additional file 1: Figure S5) and cell morphology of MB001Δ*ftsR* (Additional file 1: Figure S6) were comparable to that of ATCC13032Δ*ftsR*, and also overexpression of *ftsR* in MB001 caused similar effects as in ATCC13032 (Additional file 1:Figure S5). Thus, the absence of the prophages CGP1, CGP2, and CGP3 had no obvious influence on the effects of *ftsR* deletion and overexpression on growth and cell morphology.

### Complementation of the Δ*ftsR* phenotype with native FtsR and homologs of *C. diphtheriae* and *M. tuberculosis*

To confirm that the growth defect and the altered cell morphology of the Δ*ftsR* mutants are caused by the *ftsR* deletion rather than by secondary mutations that might have occurred during construction of the mutants or polar effects on downstream genes, complementation experiments were performed with plasmid pAN6-*ftsR* and two variants thereof. Plasmid pAN6-*ftsR*-Strep encodes an FtsR variant with a carboxyterminal linker sequence (AS) followed by a Streptag-II (WSHPQFEK). Plasmid pAN6-*ftsR*-short encodes an FtsR variant shortened by 28 amino acids at the N-terminus, which is now MSIGV. An alternative start codon was found here, based on the amino acid sequence alignment reported above (Additional file 1: Figure S1). As shown in Fig. 3b, the growth defect of ATCC13032Δ*ftsR* pAN6 could partially be reversed by pAN6-*ftsR* and pAN6-*ftsR*-Strep, but not by pAN6-*ftsR*-short. This suggests that the longer FtsR variant composed of 256 amino acid residues represents the active protein and that its function is not disturbed by a C-terminal Streptag-II. Furthermore, the morphological changes of ATCC13032Δ*ftsR* could be fully reversed by pAN6-*ftsR*. When the complementation was performed with strain MB001Δ*ftsR*, a full reversal of the growth defect and of the morphological changes was observed for MB001Δ*ftsR* pAN6-*ftsR* without IPTG induction (Additional file 1:Figures S5, S6). These results confirm that *ftsR* deletion causes the growth defect and the morphological changes of the corresponding mutants.

Due to the high conservation of the *ftsR* locus in different actinobacterial species, we assumed a similar function of the homologous genes and proteins. To test this hypothesis, we expressed *ftsR* homologs of two pathogenic relatives, CDC7B_1201 of *Corynebacterium diphtheriae* C7 and Rv1828 of *Mycobacterium tuberculosis* H37Rv, in the *C. glutamicum* MB001Δ*ftsR* strain using pAN6-based expression plasmids, and monitored growth and cell morphology. While this manuscript was in preparation, initial biochemical and structural studies of Rv1828 were published [40], which will be discussed alongside our results. With pAN6-CDC7B_1201 almost full reversal of the growth defect and of the morphological changes was achieved by basal expression without IPTG induction (Additional file 1: Figure S6). As previously observed for overexpression of *C. glutamicum ftsR*, stronger induction of CDC7B_1201 gene expression had a negative effect on growth (data not shown). With pAN6-rv1828, the best complementation was achieved in the presence of 100 μM IPTG (Additional file 1: Figure S6). Whereas a full reversal to wild-type morphology was observed, growth was improved with pAN6-rv1828 but did not reach the wild-type characteristics. These results strongly suggest that the FtsR homologs of *Actinobacteria* have similar or identical functions and target genes.

### Transcriptome comparison of the ATCC13032Δ*ftsR* mutant with its parent wild type

In order to elucidate the effects of *ftsR* deletion on *ftsZ* expression, but also on global gene expression as a first step towards definition of the FtsR regulon, transcriptome comparisons of the ATCC13032Δ*ftsR* mutant versus the parent wild type were performed using DNA microarrays and RNA isolated from cells cultivated in glucose minimal medium and harvested in the exponential growth phase. 34 genes showed a ≥ 2-fold increased mRNA level and 18 genes a ≥ 2-fold decreased mRNA level in the Δ*ftsR* mutant (Additional file 1: Table S4). Both the upregulated and the downregulated genes covered a broad and heterogeneous range of cellular functions. Within the group of genes showing a decreased mRNA level, the *ftsZ* gene with an mRNA ratio of 0.35 was the most prominent one. A reduced expression of *ftsZ* in the Δ*ftsR* mutant, combined with the fact that FtsR was enriched with the *ftsZ* promoter region, hints at a possible transcriptional activator function for FtsR. The group of upregulated genes included *oppC* and *oppD* encoding components of a putative ABC-type peptide transport system, four *prp* genes involved in propionate or propionyl-CoA catabolism, seven genes located in the CGP3 prophage region, which in total extends from cg1890 to cg2071, and a cluster of eight neighboring genes (cg0830 – cg0838) including those for an ABC-type trehalose uptake system. Interestingly, genome-resequencing revealed that the genome region comprising cg0830-cg0838 was amplified, which presumably caused the increased mRNA levels of these genes in the transcriptome analysis (Supplementary results, Additional file 1: Table S5, Additional file 1: Figure S7).

### Effect of *ftsR* deletion on *ftsZ* promoter activity and FtsZ distribution

**Fig. 4:**
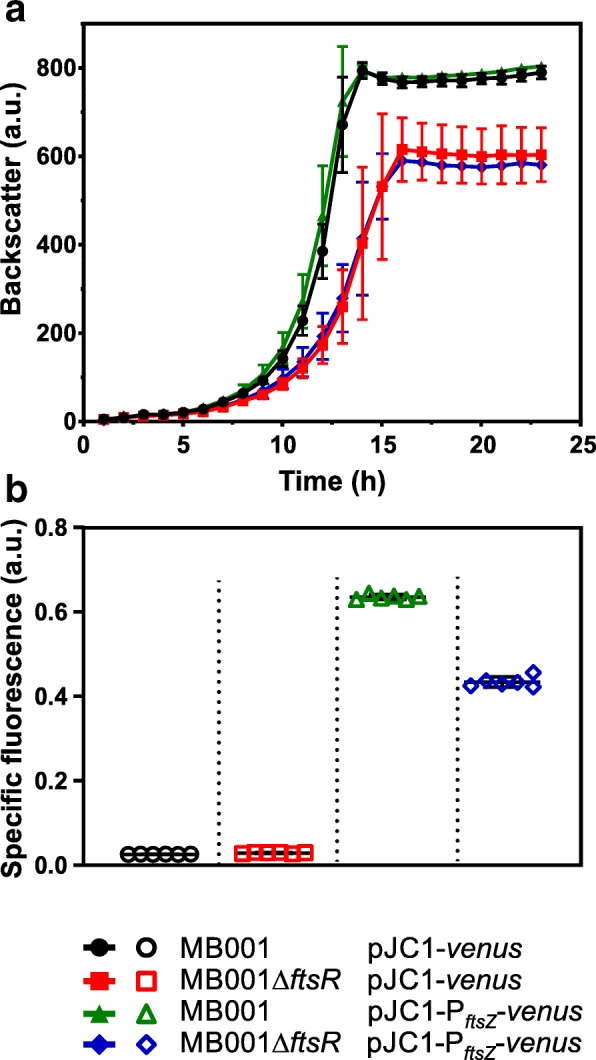
Influence of the *ftsR* deletion in *C. glutamicum* MB001 on the expression of the *venus* reporter gene under control of the *ftsZ* promoter. The indicated strains were cultivated in CGXII medium with 2% (w/v) glucose and 25 μg/mL kanamycin in a BioLector with automated measurement of Venus fluorescence and of cell density as backscatter (**a**). Specific fluorescence represents the ratio of fluorescence and backscatter at 20 h cultivation (**b**). Average values and standard deviations of three biological replicates are shown (**a**). **b** shows the result of six biological replicates

As an in vivo approach to demonstrate transcriptional activation of *ftsZ* expression by FtsR, a transcriptional fusion of the *ftsZ* promoter to a reporter gene encoding the mVenus protein was constructed resulting in plasmid pJC1-P_*ftsZ*_-*venus*. The reporter plasmid and, as negative control, the parent plasmid pJC1-*venus*-term were used to transform strains MB001 and MB001Δ*ftsR* and *ftsZ* promoter activity was measured as cell-specific fluorescence (defined as ratio of fluorescence and backscatter). As shown in Fig. 4, the lack of *ftsR* caused a significantly lower specific fluorescence, supporting activation of *ftsZ* expression by FtsR. Comparable results were obtained with strains ATCC13032 and ATCC13032Δ*ftsR* carrying pJC1-P_*ftsZ*_-*venus* (Additional file 1: Figure S8).

To test whether the lack of FtsR influences the distribution of FtsZ within the cell, we generated derivatives of ATCC13032 and ATCC13032Δ*ftsR* with a chromosomal insertion of a second copy of *ftsZ* fused in frame to the coding sequence of the fluorescent protein mVenus under control of the native *ftsZ* promoter. Both strains were cultivated in CGXII minimal medium with glucose as carbon source and analyzed by fluorescence microscopy (Additional file 1:Figure S9). In the strain lacking FtsR, the frequency as well as the intensity of the FtsZ rings was reduced. This may be caused by a reduced availability of FtsZ within the cells, which might contribute to the morphological phenotype of the *ftsR* deletion mutant.

### Genome-wide profiling of in vivo FtsR binding sites

**Fig. 5:**
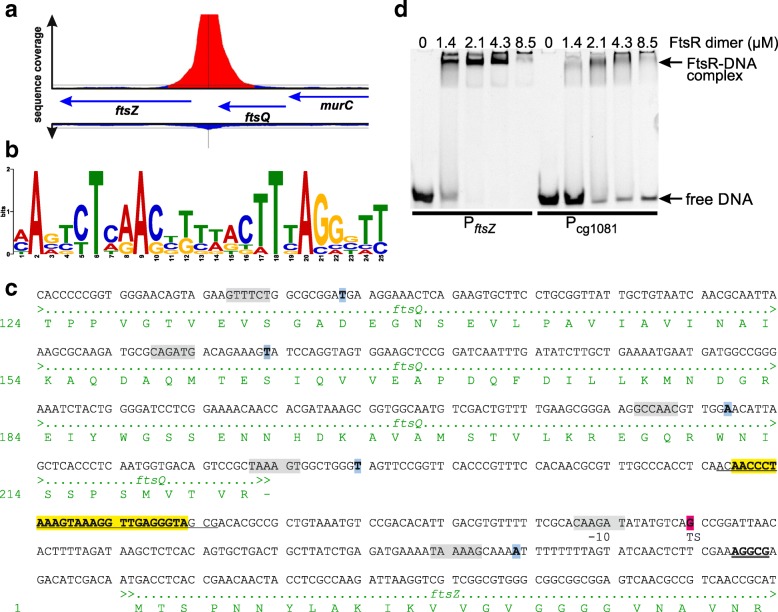
Binding of FtsR to the *ftsZ* promoter. **a** Enrichment of the *ftsZ* promoter region in the ChAP-Seq experiment with FtsR-Strep. The negative control is presented below the genes. **b** FtsR consensus DNA-binding motif identified by the MEME software [41] using the nine DNA regions with the highest coverage in the ChAP-Seq experiment with FtsR-Strep. **c** DNA sequence of the *ftsZ* promoter region including parts of the coding regions of *ftsQ* and *ftsZ* and the corresponding amino acid sequences. The ribosome binding site of *ftsZ* is double underlined. The transcriptional start site identified by RNA-Seq [29] in strain ATCC13032 is highlighted in magenta, the transcriptional start sites identified by primer extension and RACE in strain ATCC13689 [19] are indicated in blue. The deduced −10 regions are shown in grey boxes. The FtsR-binding site identified in this work is highlighted in yellow. The 30-bp region used for EMSAs with purified FtsR-Strep is underlined. **d** In vitro DNA-binding studies with FtsR-Strep. Purified FtsR-Strep was incubated in the indicated concentrations with a constant concentration (1 μM) of a 30-bp double-stranded oligonucleotide covering the predicted FtsR-binding site in the *ftsZ-*promoter region. The mixture was then analyzed by electrophoresis using a non-denaturing 15% (w/v) polyacrylamide gel. As negative control, a DNA fragment of the promoter region of cg1081 was used (1 μM)

With the aim to identify the in vivo binding site of FtsR in the *ftsZ* promoter region and to identify further target genes of FtsR, a ChAP-Seq experiment was performed. In vivo formaldehyde-crosslinked FtsR-Strep-DNA complexes were purified by StrepTactin-affinity chromatography and the isolated DNA fragments were sequenced. Nine peaks with a sequencing coverage above 2000 were detected, which is at least 50-fold higher than the background noise signal observed for the entire genome with a 40-fold coverage (Table 1, Fig. 5a, Additional file 1: Figure S10). The nine peaks were analyzed with respect to their chromosomal location and the mRNA level of the neighboring genes (Table 1). Based on the mRNA ratios observed in the transcriptome comparison of *C. glutamicum* ATCC13032Δ*ftsR* vs. ATCC13032, three potentially FtsR-activated genes (*ftsZ*, cg0852, cg2477) and five potentially FtsR-repressed genes (*cop1*-cg3181-cg3180, *phoC,* cg0838) were identified using mRNA ratio (Δ*ftsR*/wild type) cutoffs of 1.5 and 0.75. A repetition of the ChAP-Seq experiment confirmed the results of the first one except that the overall coverage was lower and the order of the peaks varied slightly (Table 1).

**Table 1:** Results of ChAP-Seq analysis with *C. glutamicum* ATCC13032Δ*ftsR*/pAN6-*ftsR*-Strep. The nine DNA regions that showed a ≥ 50-fold higher coverage than the entire genome (coverage 40) in the ChAP-Seq analysis with FtsR-Strep were analyzed with respect to the location of the peak within the genome and the mRNA levels of the genes in this region. Based on that, a prediction was made which genes could be potential FtsR targets. The arrows indicate whether the genes are encoded on the leading (→) or lagging strand (←) within the genome. Graphical representations of selected peaks as derived from the ChAP-Seq analysis are shown in Additional file 1: Figure S10

No.^a^	Coverage^a^	Peak position			DNA microarray 13032Δ*ftsR*/13032			Potential FtsR target genes
		in gene	downstream 3′-end of	upstream of	Gene	mRNA ratio	*p*-value	
1 (1)	28,811 (4781)	–	cg2478 (NCgl2178, penicillin binding protein) ←	cg2477 (NCgl2177, conserved hypothetical protein) ←	cg2478	0.75	0.11	cg2477 potentially activated by FtsR
					cg2477	0.24	0.01	
2 (5)	22,757 (934)	–	–	cg3182 (NCgl2777, *cop1*, trehalose corynemycolyl transferase) ←	cg3182	1.70	0.20	*cop1*-cg3181-cg3180 potentially repressed by FtsR
					cg3181	2.30	0.05	
					cg3180	1.91	0.05	
				cg3181 (NCgl2776, put. Secreted protein) ← cg3180 (NCgl2775, put. Secreted protein) ← cg3183 (NCgl2778, put. transposase) →	cg3183	n.a.	n.a.	
3 (2)	4437 (1556)	–	cg2367 (NCgl2076, *ftsQ*, cell division septal protein) ←	cg2366 (NCgl2075, *ftsZ*, cell division GTPase) ←	cg2367	1.20	0.02	*ftsZ* activated by FtsR
					cg2366	0.35	0.02	
4 (11)	3899 (373)	cg0470 (NCgl0381, *htaB*, heme transport-associated protein) →	cg0469 (NCgl0380, *hmuV*) →	cg0471 (NCgl0382, *htaC,* heme-transport associated protein) →	cg0469	1.41	< 0.01	–
					cg0470	1.45	0.09	
					cg0471	1.55	0.03	
5 (12)	3836 (365)	–	–	cg0852 (NCgl0712, conserv. Hypoth. protein) ←	cg0852	0.62	0.01	cg0852 potentially activated by FtsR
				cg0853 (NCgl0713, conserv. Hypoth. protein) → cg0854 (NCgl0714, *pmmA*, phosphomannomutase) → cg0855 (NCgl0715, conserv. Hypoth. protein) → cg0856 (NCgl0716, mannose-6-phosphate isomerase) →	cg0853	1.03	0.29	
					cg0854	0.89	0.25	
					cg0855	1.18	0.08	
					cg0856	1.09	0.03	
6 (3)	3630 (1529)	–	cg3392 (*oxiE*, *idhA2*, NCgl2958) → (myo-inositol 2-dehydrogenase)	cg3393 (NCgl2959, *phoC*, secreted cell-wall-associated phosphatase) →	cg3392	1.25	0.03	*phoC* potentially repressed by FtsR
					cg3393	1.60	0.03	
7 (15)	2871 (315)	cg2695 (NCgl2368, ATPase of ABC transporter) ←	cg2697 (*ssb*, NCgl2370, single-strand DNA-binding protein) ←	cg2694 (NCgl2367, put. Phosphodiesterase, nucleotide pyrophosphatase) ← cg2693 (NCgl2366, put. Phosphodiesterase, nucleotide pyrophosphatase) ←	cg2693	1.31	0.03	–
					cg2694	1.29	0.05	
					cg2695	1.06	0.42	
					cg2697	1.04	0.24	
8 (6)	2355 (858)	–	cg2518 (NCgl2213, put. Secreted protein) → cg2519 (NCgl2214, conserv. Hypoth. protein) ← cg2520 (NCgl2215, hypoth. protein) ← cg2521 (NCgl2216, *fadD15*, long chain fatty acid CoA ligase) ←	–	cg2518	0.81	0.18	–
					cg2519	0.49	0.03	
					cg2520	0.92	0.45	
					cg2521	0.91	0.43	
9 (8)	2248	cg0839 (NCgl0701) ← (hypoth. protein)	cg0840 (NCgl0702, conserv. Hypoth. protein) ←	cg0838 (NCgl0700, ATP-depend. helicase) ←	cg0838	4.61	0.03	cg0838 potentially repressed
					cg0839	2.45	0.16	by FtsR
					cg0840	2.45	0.19	

^a^ The numbers in brackets represent the result of a second, independent experiment. Please note that the overall coverage was lower in the second experiment

The genes cg0852 and cg2477 encode conserved proteins of unknown function. The putative operon *cop1*-cg3181-cg3180 encodes three secreted proteins [42]. The Cop1 protein (also termed Csp1) was shown to function as mycolyltransferase involved in the conversion of trehalose monocorynomycolate to trehalose dicorynomycolate [42, 43]. The protein encoded by *phoC* was proposed to function as cell-wall-associated phosphatase [44, 45] and is induced under phosphate-limiting conditions [46]. The cg0838 gene is part of the DNA region found to be amplified in the ATCC13032Δ*ftsR* mutant but had a higher mRNA ratio than the neighboring genes that were also amplified (Additional file 1: Table S5), pointing to a possible repression by FtsR. The large Cg0838 protein (179 kDa) is proposed to function as an ATP-dependent helicase and contains a unique C-terminal domain including a metal-binding cysteine cluster.

The search for a common DNA sequence motif in the nine sequences with a coverage above 2000 using the MEME software [41] revealed the motif shown in Fig. 5b. The motif was identified in eight of the nine sequences except for the sequence with a coverage of 2871. When looking at the corresponding motif in the *ftsZ* promoter region, it forms an imperfect 25-bp inverted repeat: AACCCTAAAGTAAAGGTTGAGGGTA. The center of this motif was located at position − 73 with respect to the transcriptional start site of *ftsZ* as determined by RNA-Seq [29]. This position is compatible with an activating function of FtsR for *ftsZ* expression (Fig. 5c). It should be mentioned that in *C. glutamicum* strain ATCC13689, five transcriptional start sites were identified for *ftsZ* by primer extension and RACE studies [19], none of which corresponds to the one identified by RNA-Seq for strain ATCC13032 [29].

**Fig. 6:**
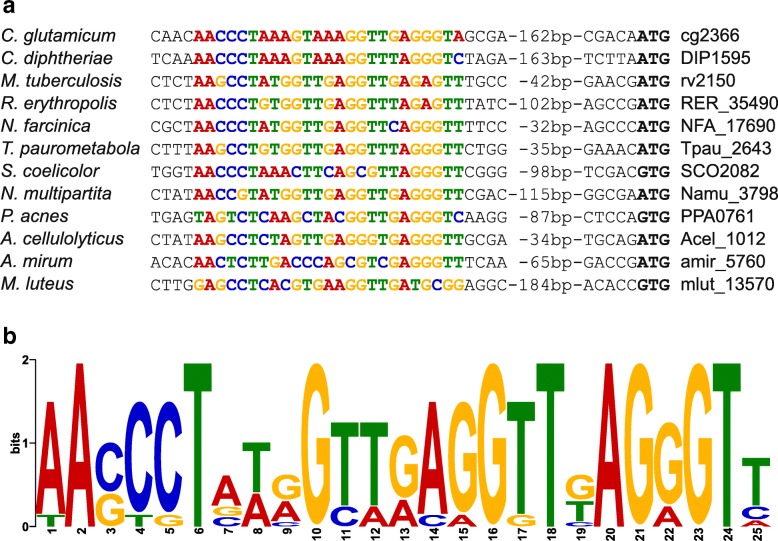
Putative FtsR binding sites in the *ftsZ* promoters of various actinobacteria. **a** Proposed FtsR binding sites in the *ftsZ* promoters of several actinobacterial species identified by sequence alignment with the FtsR binding site in the *C. glutamicum ftsZ* promoter. The respective *ftsZ* locus tags are given on the right. The proposed binding motifs are shown as colored letters. The annotated start codons of the FtsZ proteins are indicated by bold letters. The distance between the proposed FtsR binding site and the start codon varies from 41 to 193 bp. **b** Consensus DNA-binding motif generated by MEME from the sequences shown in **a** using default parameters

The DNA-binding motif shown above was used to search for similar motifs in the *ftsZ* promoter regions of other actinobacterial species possessing FtsR homologs using Clustal Omega [47]. All analyzed promoters contained a similar motif (Fig. 6a), supporting the assumption that regulation of *ftsZ* expression by FtsR homologs is a conserved mechanism in FtsR-containing *Actinobacteria*. The motif generated from the proposed binding sites also represents a 25 bp inverted repeat (Fig. 6b), which is very similar to the one derived from the *C. glutamicum* sequences enriched by ChAP-Seq with FtsR (Fig. 5b).

### In vitro binding of purified FtsR to the proposed binding motif in the *ftsZ* promoter region

To test whether purified FtsR is able to bind to the proposed binding motif in the *ftsZ* promoter region, FtsR-Strep was overproduced in *C. glutamicum* ATCC13032Δ*ftsR* using plasmid pAN6-*ftsR*-Strep and purified using StrepTactin-Sepharose (Additional file 1: Figure S11A). As described above, FtsR-Strep could complement the growth defect of the ATCC13032Δ*ftsR* mutant to the same extent as native FtsR, indicating that the StrepTag did not negatively influence FtsR activity. Size-exclusion chromatography of affinity-purified FtsR-Strep (calculated mass 28.5 kDa) and comparison to standard proteins indicated that the protein forms a dimer (Additional file 1: Figure S11B). This is in line with the dimeric state of the homologous protein Rv1828 [40]. Purified FtsR-Strep was able to completely shift a 30-bp double-stranded oligonucleotide covering the predicted FtsR-binding site in the *ftsZ* promoter region at a 2-fold molar excess of the dimeric protein (Fig. 5d). In contrast, a control 30-bp double-strand-oligonucleotide derived from the promoter region of cg1081 was incompletely shifted even at an 8.5-fold molar excess of dimeric FtsR-Strep. The specificity of this interaction was further tested using a competition-EMSA (Additional file 1: Figure S12). A Cy3-labelled 271-bp DNA fragment covering the *ftsZ* promoter region including the FtsR binding site was incubated with FtsR protein and increasing concentrations of either specific (same fragment as above) or unspecific unlabeled competitor DNA. Only the specific competitor DNA reversed the shift of the labelled DNA fragment by FtsR, supporting its specific binding to the *ftsZ* promoter region.

### FtsR-independent expression of FtsZ

**Fig. 7:**
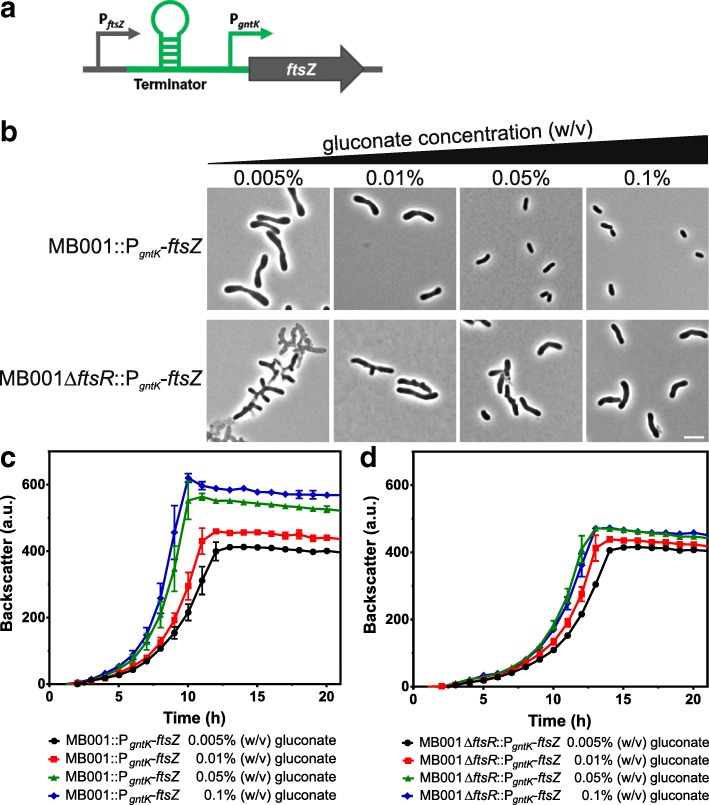
Promoter exchange of *ftsZ* and growth of the resulting strains. **a** Strains with FtsR-independent *ftsZ* expression were constructed using a DNA fragment with a terminator sequence and the gluconate-inducible *gntK* promoter, which was inserted between the native *ftsZ* promoter and the *ftsZ* start codon in the chromosomes of MB001 and the MB001Δ*ftsR* mutant. **b-d** Effect of different gluconate concentrations on cell morphology (**b**) and growth (**c, d**) of the promoter exchange strains MB001::P_*gntK*_-*ftsZ* and MB001Δ*ftsR*::P_*gntK*_-*ftsZ*. The two strains were first pre-cultivated in BHI medium supplemented with 0.1% (w/v) gluconate to induce *ftsZ* expression by P_*gntK*_. The second pre-cultivation was performed in CGXII medium with 2% (w/v) glucose supplemented with the indicated gluconate concentrations. The main cultures were then performed in media having the same composition as the ones for the second pre-cultivation. **b** Microscopic pictures of cells from the stationary phase. The scale bar represents 5 μm. **c, d** The growth experiments show mean values and standard deviations of three biological replicates

It is known from previous studies that differences in *ftsZ* expression cause aberrant cell morphology and FtsZ localization in *C. glutamicum* [19]. In order to test whether the altered cell morphology and the growth defect of the Δ*ftsR* mutant are solely caused by the differences in *ftsZ* expression, we constructed strains in which the expression of *ftsZ* is independent of FtsR and controlled by the gluconate-inducible *gntK* promoter of *C. glutamicum* [19, 37, 48]. A DNA fragment containing a terminator sequence and the *gntK* promoter was inserted between the native *ftsZ* promoter and the ribosome binding site of *ftsZ* within the chromosomes of *C. glutamicum* MB001 and MB001Δ*ftsR* by double homologous recombination (Fig. 7a). The promoter exchange strains MB001::P_*gntK*_-*ftsZ* and MB001Δ*ftsR*::P_*gntK*_-*ftsZ* showed growth in CGXII medium containing gluconate, but not in medium containing only glucose or sucrose, confirming gluconate-inducible *ftsZ* expression (Additional file 1: Figure S13).

In order to analyze the consequences of different *ftsZ* expression levels on growth and morphology, cells were cultivated in CGXII medium with 2% (w/v) glucose supplemented with 0.005, 0.01, 0.05, and 0.1% (w/v) gluconate. Strain MB001::P_*gntK*_-*ftsZ* showed enlarged cells with thickened poles at the lowest gluconate concentrations and reverted back to wild-type morphology at 0.1% (w/v) gluconate (Fig. 7b). Strain MB001Δ*ftsR*::P_*gntK*_-*ftsZ* displayed strongly branched cells at the two lowest gluconate concentrations and even at 0.1% (w/v) gluconate the cells were still enlarged compared to the *ftsR*-positive strain (Fig. 7b). With respect to growth, the two strains showed quite similar growth behavior at the lowest gluconate concentration. Increased gluconate concentrations strongly improved growth of the *ftsR*-positive strain (Fig. 7c), whereas growth of the *ftsR*-negative strain was only marginally increased (Fig. 7d).

Overall, these results demonstrate the importance of an appropriate FtsZ level for normal cell morphology and growth, but they also show that the branched cells of the Δ*ftsR* mutant are not solely caused by reduced *ftsZ* expression, but presumably also by altered expression of other genes as a consequence of the *ftsR* deletion. Similarly, the growth defect of the Δ*ftsR* mutant cannot be rescued simply by increased expression of *ftsZ* and thus involves further genes. These could either be direct target genes of FtsR or genes whose expression is indirectly influenced by the absence of FtsR. In additional experiments, the promoter exchange strains with FtsR-independent *ftsZ* expression were also used to confirm transcriptional activation of *ftsZ* expression by FtsR using the reporter plasmid pJC1-P_*ftsZ*_-*venus* (see Supplemental results and Additional file 1: Figure S14).

## Discussion

The tubulin-like GTPase FtsZ plays the key role in bacterial cell division [49]. In this study, we identified the transcriptional regulator FtsR that functions as activator of *ftsZ* expression in *C. glutamicum*, representing a novel control mechanism of actinobacterial cytokinesis. FtsR was initially enriched by DNA affinity chromatography with the *ftsZ* promoter region. In vivo binding of FtsR to the *ftsZ* promoter was confirmed by ChAP-Seq experiments and purified FtsR was shown to bind to a 30 bp-DNA fragment covering the proposed DNA-binding site of FtsR, which represents an imperfect 25-bp inverted repeat. Evidence for the activation of *ftsZ* expression by FtsR came from transcriptome analysis, where the *ftsZ* mRNA level was significantly reduced in the Δ*ftsR* mutant, and reporter gene studies showing reduced activity of the *ftsZ* promoter in the Δ*ftsR* background.

Bioinformatic analyses revealed that FtsR belongs to the MerR superfamily of transcriptional regulators, which mostly function as activators, responding to a vast diversity of stimuli including oxidative stress, xenobiotics, and metal ion stress [50, 51]. MerR-type regulators typically bind as homodimers to inverted repeats in the promoter region of their target genes. The binding sites can be located between the − 10 and − 35 regions, which then show a spacing of 19–20 bp rather than the optimal 16–18 bp. This hampers transcription because the − 10 and − 35 regions are located on opposite sides of the DNA strand [51]. A well-described mechanism of transcriptional activation by regulators of the MerR superfamily is thought to rely on a conformational change of the N-terminal DNA-binding region after recognition of a stimulus by the C-terminal domain, which causes a twist of the bound DNA molecule in a manner that the − 10 and − 35 regions are rearranged, enabling RNA polymerase to bind and initiate transcription [52–55]. FtsR eluted as dimer during gel filtration and the binding site identified in the *ftsZ* promoter region was found to be an inverted repeat, which was located at position − 61 to − 85 upstream of the *ftsZ* transcriptional start site identified by RNA-Seq [29] rather than between the − 10 and − 35 regions. This position is compatible with an activating function of FtsR, but might involve a different mechanism of transcriptional activation than the one described above.

It should be mentioned that in *C. glutamicum* strain ATCC13689 five transcriptional start sites were identified for *ftsZ* by RACE and primer extension experiments [19], none of which corresponds to the one identified by RNA-Seq for strain ATCC13032 [29]. Further studies are required to test for the presence of additional transcriptional start sites of the *ftsZ* gene in strain ATCC13032, in which the FtsR binding site might be located between the − 10 and − 35 regions. Multiple promoters for *ftsZ* expression appear to be the rule in various bacteria such as *M. tuberculosis* [22, 24], *E. coli* [23], *Streptomyces coelicolor* A3 [2] [56], or *Streptomyces griseus* [57]. They allow a dynamic adjustment of the FtsZ levels according to different stimuli and probably make the cells more robust with respect to spontaneous mutations in the *ftsZ* promoter region.

FtsR was found to be conserved in many different families of *Actinobacteria*, including the genus *Mycobacterium*. In a ChIP-Seq experiment with the FtsR homolog Rv1828 of *M. tuberculosis*, the promoter region of the *ftsZ* gene was the top target [58–60]. Using the proposed binding motif of FtsR (AACCCTAAAGTAAAGGTTGAGGGTA) as template, we identified a very similar sequence (AACtCTAAgcctAtGGTTGAGGtTt) in the *ftsZ* promoter of *M. tuberculosis*, the region which also showed by far the highest coverage in the ChIP-Seq experiment [59]. Here the motif was located exactly between the − 10 and − 35 regions of the transcriptional start site P1, which is located at position − 43 relative to the *ftsZ* translational start [24]. The binding motif proposed for Rv1828 in a recent study is different but overlaps partially with our motif [40]. Mutational analysis of this binding motif by EMSAs revealed that binding of Rv1828 is not influenced when the specific part of the motif is altered that does not overlap with our proposed binding motif [40]. This, together with the strong conservation of our motif among many species, suggests that the motif proposed by us is more likely the correct one. Our results obtained for FtsR and Rv1828 together with the recently published characterization of Rv1828 [40] strongly support the notion that FtsR and its actinobacterial homologs are involved in transcriptional regulation of *ftsZ*. In line with this, the *ftsZ* promoter regions of various actinobacterial genera were found to contain DNA sequence motifs similar to the ones determined for FtsR and Rv1828 (Fig. 6).

Deletion and overexpression of *ftsR* led to growth inhibition and an altered cell morphology. The deletion phenotype could be reversed by plasmid-based expression of *ftsR* or the *ftsR* homologs of *C. diphtheriae* and *M. tuberculosis* (Fig. 3, Additional file 1:Figures. S2, S3, S4). The fact that many Δ*ftsR* cells and also *ftsR* overexpression cells contained multiple septa indicates that cell division is disturbed. The studies with the promoter exchange strains, in which *ftsZ* expression was controlled by the gluconate-inducible *gntK* promoter, revealed that growth behavior and morphology of the Δ*ftsR* mutant are not solely caused by altered *ftsZ* expression. Despite comparable *ftsZ* expression, the Δ*ftsR* strain grew much worse than the *ftsR*-positive reference strain (Fig. 7c, d) and the morphology of the Δ*ftsR* strain differed from the *ftsR*^+^ strain, in particular by the formation of branched cells (Fig. 7b). This suggests that i) FtsZ alone is not the cause for the branching phenotype, and ii) there must be some other, FtsR-dependent gene that causes enlarged cells even in the presence of sufficient FtsZ.

Potential further target genes of FtsR were deduced by combining ChAP-Seq and transcriptome results. The target with the most obvious relation to the altered cell morphology of the Δ*ftsR* mutant was the *cop1*-cg3181-cg3182 gene cluster. These three genes encode secreted proteins and Cop1 (Csp1), a mycolyltransferase converting trehalose monocorynomycolate to trehalose dicorynomycolate. The mycolyl transferase function was located in the N-terminal portion of Cop1 [43]. A *C. glutamicum cop1* deletion mutant had an altered cell morphology characterized by enlarged, club-shaped cells and it was speculated that the C-terminal part of Cop1 plays a role in cell shape formation [42]. In the case of *cop1*, the FtsR-binding site (AAGTCTAAAGTTGAACTTAAGATTG) starts downstream of the − 35 region and ends downstream of the − 10 region (TAAGAT). The transcriptome data suggest a repression of *cop1* by FtsR and the morphology of the cells overexpressing *ftsR* (Fig. 3c, Additional file 1: Figure S3) resemble that of cells lacking functional *cop1* [42]. Further studies are required to confirm the influence of FtsR on the expression of the *cop1*-cg3181-cg3182 gene cluster and to identify further target genes and their function.

Regarding the physiological function of FtsR, a possible role could be to serve as a regulatory mechanism that controls cell division in response to the nutritional status of the cell. In recent years, several studies have revealed metabolic enzymes to directly influence cell division by modulating the activity of FtsZ [61]. Transcriptional regulation, such as shown here for FtsR, is another means to influence FtsZ activity. The fact that FtsR activates *ftsZ* expression suggests that FtsR signals favorable nutritional conditions with a high cell division rate and thus a high FtsZ demand. As the *ftsR* gene and its homologs in other *Actinobacteria* are always located downstream of the *odhI*/*garA* gene, a functional link to OdhI/GarA might exist. OdhI/GarA controls the metabolic flux at the 2-oxoglutarate node of metabolism, which is particularly relevant for nitrogen assimilation. Our preliminary attempts to find a potential ligand of FtsR were not successful and the identification of the stimulus controlling FtsR activity is ultimately required to understand the function of this regulator.

## Conclusion

In this study, we identified and characterized FtsR as the first transcriptional regulator of FtsZ described for *C. glutamicum*. Deletion or overexpression of *ftsR* had severe effects on growth and cell morphology, underlining the importance of FtsR for correct cell division. The fact that FtsR activates *ftsZ* expression suggests that FtsR might sense favorable nutritional conditions that allow for a high cell division rate and therefore require high FtsZ levels. In summary, our findings provide a first insight into transcriptional regulation of cell division in Actinobacteria.

## Methods

### Bacterial strains, plasmids and growth conditions

Bacterial strains and plasmids used in this study are listed in Additional file 1: Table S2. The *C. glutamicum* type strain ATCC13032 or its prophage-free variant *C. glutamicum* MB001 were used as wild type or reference strain, as indicated. The parental strain ATCC13032 (DSM No. 20300) was obtained from the German Collection of Microorganisms and Cell Cultures GmbH (DSMZ, Braunschweig, Germany). The prophage-free strain MB001 is from in-house stock and can also be obtained from the DSMZ (DSM No. 102070). For growth experiments, *C. glutamicum* was precultivated for 6–8 h at 30 °C and 170 rpm in 5 mL BHI medium (BD Bacto™ Brain Heart Infusion, Becton Dickinson and Company, Heidelberg, Germany). The cells were harvested by centrifugation, washed with phosphate-buffered saline (PBS, 137 mM NaCl, 2.7 mM KCl, 4.3 mM Na_2_HPO_4_, 1.4 mM KH_2_PO_4_, pH 7.3) and used as inoculum for a second preculture in 20 mL CGXII minimal medium [62] containing 30 mg/L 3,4-dihydroxybenzoate as iron chelator and, if not stated otherwise, 2% (w/v) glucose as carbon source. This preculture was incubated overnight at 30 °C and 120 rpm and after harvesting the cells were washed in PBS and used for inoculation of the main culture, using again CGXII medium with 30 mg/L 3,4-dihydroxybenzoate and 2% (w/v) glucose. Growth experiments were either performed in 100 mL shake flasks with 20 mL medium (initial optical density at 600 nm (OD_600_) of 1.0) that were shaken at 30 °C and 120 rpm or in 48-well FlowerPlates® (m2p-labs GmbH, Baesweiler, Germany) with a culture volume of 800 μL (initial OD_600_ of 0.5) that were shaken in a BioLector (m2p-labs GmbH, Baesweiler, Germany) at 30 °C and 1200 rpm. Growth was monitored as cell density by determining either OD_600_ (shake flasks) or as scattered light at 620 nm in the BioLector [63], which is termed “backscatter” throughout this study. For growth experiments with promoter exchange strains where expression of the *ftsZ* gene was under control of the *gntK* promoter, the first pre-culture was supplemented with 0.1% (w/v) gluconate and the second pre-culture were supplemented with 0.01% (w/v) gluconate and 1.99% (w/v) glucose as carbon source. The gluconate concentration of the main culture is indicated for each experiment. For cloning purposes, *Escherichia coli* DH5α was used and routinely cultivated at 37 °C in lysogeny broth (LB) [64]. When required, the media were supplemented with 25 μg/mL kanamycin or 10 μg/mL chloramphenicol for *C. glutamicum* or with 50 μg/mL kanamycin or 34 μg/mL chloramphenicol for *E. coli*.

### Recombinant DNA work and construction of insertion and deletion mutants

Routine methods such as PCR, DNA restriction and ligation, Gibson assembly and transformation were performed using standard protocols [64–67]. Phusion Green High Fidelity DNA Polymerase (Thermo Fisher Scientific Inc., Rockford, IL, USA) was used for cloning purposes. For all other PCRs, either the KAPA2G Fast ReadyMix PCR Kit (Kapa Biosystems, Wilmington, USA) or DreamTaq DNA Polymerase (Thermo Fisher Scientific Inc., Rockford, IL, USA) were used. The oligonucleotides used in this study are listed in Additional file 1: Table S3 and were purchased from Eurofins Genomics GmbH (Ebersberg, Germany). DNA sequencing was performed by Eurofins Genomics GmbH. The Δ*ftsR* mutants of *C. glutamicum* and the promoter exchange strains carrying a DNA fragment with a transcription terminator and the *gntK* promoter inserted into the chromosome between the native *ftsZ* promoter and the *ftsZ* coding region were constructed via a two-step homologous recombination protocol as described [37, 68, 69] using plasmids pK19*mobsacB*-Δ*ftsR* and pK19*mobsacB*-P_*gntK*_*-ftsZ*. The strains ATCC13032::*ftsZ*-venus and ATCC13032Δ*ftsR*::*ftsZ*-venus were constructed by chromosomal insertion of pK18*mob-ftsZ-venus* by single homologous recombination. In all tested clones of both strains, the plasmid did not insert into the intergenic region between cg1121 and cg1122, but in the *ftsZ* region. As the insertion plasmid was constructed in such a way that these mutants also carry one native copy of *ftsZ* and one fused to *venus*, these strains were used for microscopy, anyway.

### Fluorescence microscopy

*C. glutamicum* cells were centrifuged and suspended in PBS containing 100 ng/mL Hoechst 33342 dye to stain DNA and 300 ng/mL Nile red to stain membranes (Sigma-Aldrich Chemie GmbH, Taufkirchen, Germany). After 10 min of incubation in the dark at room temperature, samples were spotted onto a glass slide covered with a thin agarose layer (1% (w/v) in TAE buffer (40 mM Tris, 1 mM Na_2_EDTA, 20 mM glacial acetic acid, pH 8)) and analyzed using a Zeiss Axio Imager M2 microscope equipped with a Zeiss AxioCam MRm camera (Carl Zeiss AG, Oberkochen, Germany). Images were acquired using a Plan-Apochromat 100×/1.40-numerical aperture phase contrast oil immersion objective and AxioVision 4.8 software (Carl Zeiss Microscopy GmbH, Jena, Germany). Hoechst 33342 fluorescence was visualized with filter set 49 and Nile red with filter set 63 HE (both Carl Zeiss AG). *C. glutamicum* strains producing the FtsZ-Venus fusion protein were directly used for microscopy without further staining.

### Purification of FtsR

FtsR-Strep was purified using *C. glutamicum* ATCC13032Δ*ftsR* carrying plasmid pAN6-FtsR-Strep. The preculture was prepared using a single colony from a fresh BHI agar plate to inoculate 20 mL BHI with 2% (w/v) glucose and 25 μg/mL kanamycin and incubated at 30 °C and 120 rpm for 6–8 h. The main culture (500 mL of the same medium in a 2 L baffled flask) was inoculated with 1 mL of the preculture and incubated overnight at 30 °C and 90 rpm. On the following morning, 50 μM isopropyl β-D-1-thiogalactopyranoside (IPTG) was added and the culture was incubated for additional 4 h at 30 °C and 120 rpm. Subsequently, the cells were harvested by centrifugation (4 °C, 30 min, 3399 *g*) and resuspended in 25 mL buffer A (100 mM Tris-HCl, 100 mM NaCl, pH 7.5) supplemented with cOmplete Mini EDTA-free protease inhibitor cocktail tablets (Roche Diagnostics GmbH, Mannheim, Germany). Cell disruption was performed by five passages through a French pressure cell using a HTU-Digi-F-Press (G. Heinemann Ultraschall- und Labortechnik, Schwaebisch Gmuend, Germany) at a pressure of 103.4 MPa (15,000 psi). Cell debris was removed by centrifugation for 20 min at 5300 *g* and 4 °C, followed by ultracentrifugation for 1 h at 84,000 *g* and 4 °C. The supernatant was used for affinity chromatography using a Strep-Tactin-Sepharose column (IBA, Göttingen, Germany) with 1 mL bed volume equilibrated with buffer W (50 mM Tris-HCl, 250 mM NaCl, pH 7.5). After the protein extract had passed, the column was washed three times with 15 mL buffer W (50 mM Tris-HCl, 250 mM NaCl, pH 7.5) and FtsR-Strep was eluted with 10 × 1 mL buffer E (buffer W with 15 mM d-desthiobiotin obtained from Sigma-Aldrich Chemie GmbH, Steinheim, Germany). Aliquots of the elution fractions were analyzed by SDS-PAGE [70] and the fractions containing FtsR-Strep were pooled and concentrated using Amicon Ultra-4 Centrifugal Filter Devices with a 10 kDa cut-off (Merck Millipore Ltd., Carrigtwohill, Cork, Ireland). FtsR-Strep was further purified by gel filtration using a Superdex™ 200 Increase 10/300 GL column attached to an ÄKTA™ pure 25 system (GE Healthcare Bio-Sciences AB, Uppsala, Sweden). The column was equilibrated with buffer W and elution was performed with a flow rate of 0.6 mL/min.

### Electrophoretic mobility shift assays (EMSAs)

Two complementary single-stranded 30-bp oligonucleotides (see Additional file 1: Table S3) covering the putative FtsR-binding motif in the *ftsZ* promoter region were annealed by heating the samples containing 10 μM Tris-HCl pH 8.0, 50 mM NaCl, 1 mM EDTA and 10 μM of each oligonucleotide to 95 °C for 5 min, followed by slow cooling to room temperature. The double-stranded oligonucleotide (final concentration 1 μM) was incubated at room temperature for 30 min with increasing concentrations (0–8.5 μM) of freshly purified dimeric FtsR protein (Additional file 1: Figure S11) in binding buffer (10 mM Tris-HCl pH 7.5, 30 mM NaCl, 1.5 mM Na_2_EDTA). A 30 bp fragment located in the cg1081 promoter region was used as negative control (Additional file 1: Table S3). After incubation, a suitable volume of the 5-fold concentrated sample buffer (0.1% (w/v) xylene cyanol, 0.1% (w/v) bromophenol blue, 20% (v/v) glycerol in 1x TBE (89 mM Tris base, 89 mM boric acid, 2 mM Na_2_EDTA)) was added and the samples were separated by native PAGE using 15% (w/v) polyacrylamide gels in 1x TBE buffer. Subsequently, the DNA was stained with SYBR® Green I Nucleic Acid Gel Stain (Invitrogen, Ltd., Paisley, UK) and photographed. The competition-EMSA was performed as described previously [71]. In brief, a 271 bp DNA-fragment covering the *ftsZ* promoter region including the binding site was amplified using oligonucleotides ftsZ_prom250_fw andCy3-ftsZ_prom_rv to generate Cy3-labelled DNA or oligonucleotides ftsZ_prom250_fw/ftsZ_prom_rv to generate unlabeled specific competitor DNA. A 260 bp DNA-fragment further upstream in the *ftsZ* promoter was amplified using the oligonucleotide-pair ftsZ_prom500_fw/ftsZ_prom250up_rv and used as unspecific competitor DNA. Purified FtsR protein was incubated with the DNA fragment(s) in a total volume of 10 μL binding buffer. After addition of loading buffer and separation by native PAGE on a 10% polyacrylamide gel, the gel was scanned using a Typhoon TrioTM scanner (GE Healthcare).

### Promoter activity studies with P_*ftsZ*_

In order to analyze transcriptional regulation of *ftsZ* by FtsR in vivo, a DNA fragment covering the *ftsZ* promoter region and the native ribosomal binding site extending from position − 494 to + 3 with respect to the *ftsZ* translational start site was fused to a DNA sequence coding for the fluorescent protein mVenus and cloned into the pJC1 vector backbone (for details see Additional file 1: Tables S4 and S5). Strains carrying their chromosomal *ftsZ* gene either under control of its native promoter or under control of the *gntK* promoter were transformed with the resulting plasmid pJC1-P_*ftsZ*_-*venus*. For complementation studies, the strains were additionally transformed with pEC-*ftsR* or pEC-XC99E as control. Growth experiments were performed in the BioLector® as described above, with additional monitoring of the *ftsZ* promoter activity by online measurement of mVenus fluorescence at an excitation wavelength of 508 nm and an emission wavelength of 532 nm.

### Chromatin affinity purification with subsequent sequencing (ChAP-Seq)

For ChAP-Seq experiments, *C. glutamicum* ATCC13032Δ*ftsR*/pAN6-FtsR-Strep and as negative control *C. glutamicum* ATCC13032Δ*ftsR*/pAN6 were cultivated as described above for FtsR purification with 10 μM instead of 50 μM IPTG. FtsR-Strep was purified as described above with the following alterations: after harvesting (15 min, 6371 *g*, 4 °C), washing with 40 mL PBS, and a second centrifugation step (15 min, 5525 *g*, 4 °C), the supernatant was discarded and the cells were suspended in 20 mL PBS containing 1% (w/v) formaldehyde and incubated at room temperature for 20 min, followed by addition of glycine to a final concentration of 125 mM and incubation for another 5 min at room temperature. The cells were washed twice with 40 mL buffer A and suspended in 20 mL buffer A with a suitable amount of cOmplete Mini EDTA-free protease inhibitor cocktail tablets and 5 mg of RNAse A (both Roche Diagnostics GmbH, Mannheim, Germany). After cell disruption using a French press as described above, the crude extract was transferred to a sonication vessel, placed in an ice bath and sonified 3 × 30 s using a Branson Sonifier 250 (G. Heinemann Ultraschall- und Labortechnik, Schwaebisch Gmuend, Germany) with a pulse length of 40% and an intensity of one to shear the genomic DNA. The sonified crude extract was used for StrepTactin affinity chromatography as described above. The FtsR-containing elution fractions were pooled, 1% (w/v) SDS was added, and the samples were incubated overnight at 65 °C, followed by proteinase K treatment (final concentration 400 μg/mL) for 3 h at 55 °C. The DNA in the samples was purified by phenol-chloroform-isoamyl alcohol extraction [72], precipitated with ethanol and sodium acetate, washed with 70% (v/v) ethanol, dried and dissolved in 50 μL deionized water. Sequencing of the DNA fragments and data evaluation were performed as described previously [73]. Genomic binding sites of FtsR were defined to have a sequence coverage that is at least 50-fold above the average genome coverage determined for the negative control strain *C. glutamicum* ATCC13032 pAN6.

### DNA microarrays

DNA microarray analysis was performed to compare the mRNA levels of the ATCC13032Δ*ftsR* mutant with the wild type. Cells were cultivated in 50 mL CGXII medium with 2% (w/v) glucose and harvested on ice in the exponential growth phase. RNA sample preparation, labelling, hybridization, and comparative transcriptome analysis were performed as described previously [74]. The full data set of this experiment has been deposited in the NCBI Gene Expression Omnibus and can be found under the GEO accession number GSE107921.

### DNA affinity purification and MALDI-ToF-MS analysis

To identify putative regulatory proteins binding to the *ftsZ* promoter region, DNA affinity chromatography was performed. A 404-bp DNA fragment, extending from position − 285 to + 96 with respect to the translational start site of *ftsZ* and including a 23 bp overhang for the addition of the biotin-tag, was amplified by PCR using the oligonucleotides AP_PftsZ_fw and AP_PftsZ_rv_bio. The resulting fragment was tagged with biotin by a second PCR using the biotinylated oligonucleotide biotin_oligo and AP_PftsZ_fw. The PCR products were purified by size exclusion chromatography using a column with 8 mL Sephacryl™ S-400 High Resolution Chromatography Media (GE Healthcare Bio-Sciences AB, Uppsala, Sweden) as bed material. Fractions containing the desired DNA fragment were pooled, concentrated to about 500 μL using an Eppendorf Concentrator plus (Eppendorf AG, Hamburg, Germany) and precipitated with ethanol. The precipitate was dissolved in 50 μL TE buffer and the DNA concentration was measured with a Colibri Microvolume Spectrophotometer (Berthold Detection Systems GmbH, Pforzheim, Germany) to assure a minimal amount of 220 pmol biotinylated DNA, which was required for the following steps. The DNA affinity chromatography was performed using Dynabeads® M-280 Streptavidin (Life Technologies AS, Oslo, Norway) as described [73]. In brief, the biotinylated DNA was coupled to the beads and incubated with crude cell extract of *C. glutamicum* ATCC13032 cells that had been cultivated in glucose minimal medium and harvested in the exponential growth phase. After several washing steps, the proteins were eluted using buffer containing 2 M NaCl. The elution fractions were subjected to protein precipitation with trichloroacetic acid (TCA, 10% (w/v) final concentration) and washed once with acetone. The precipitated protein was dissolved in 20 μL Tris-HCl buffer pH 7.5 and analyzed by SDS-PAGE [70] using a 12% Mini-PROTEAN® TGX™ gel (Bio-Rad Laboratories, Inc., Hercules, CA, USA). The gel was stained with GelCode® Blue Stain Reagent (Thermo Fisher Scientific Inc., Rockford, IL, USA). Proteins enriched with the promoter region of *ftsZ* were identified by peptide mass fingerprinting after tryptic in-gel digestion of the excised bands of the polyacrylamide gel followed by MALDI-ToF-MS using an Ultraflex III TOF/TOF mass spectrometer (Bruker Daltonics, Bremen, Germany) as described [75, 76].

## Additional file

Additional file 1: Contains additional methods, results, figures and tables. (PDF 3699 kb)

## Data Availability

The microarray data has been deposited in the GEO database under GSE107921 (https://www.ncbi.nlm.nih.gov/geo/query/acc.cgi?acc=GSE107921). All other data generated or analysed during this study are included in this published article and its supplementary information files. All plasmids and strains used in this study are available from the corresponding author.

## Abbreviations

BHI: Brain Heart Infusion (complex medium for *C. glutamicum*)
CGP1, CGP2, CGP3: *C. glutamicum* prophages 1, 2 and 3
CGXII: minimal medium for *C. glutamicum*
IPTG: Isopropyl β-D-1-thiogalactopyranoside
LB: Lysogeny broth
OD_600_: Optical density at 600 nm
PBS: Phosphate buffered saline
TCA: Trichloroacetic acid
