# Supplementary material for: The conserved actinobacterial transcriptional regulator FtsR controls expression of ftsZ and further target genes and influences growth and cell division in Corynebacterium glutamicum
Source: BMC Microbiol. 2019 Aug 5;19:179. doi: 10.1186/s12866-019-1553-0 (PMC6683498; doi:10.1186/s12866-019-1553-0)
Supplement: Supplementary file 1 — Contains additional methods, results, figures and tables. (PDF 3699 kb) [file 12866_2019_1553_MOESM1_ESM.pdf]

Supplemental Material to

The conserved actinobacterial transcriptional regulator FtsR controls expression of *ftsZ* and further target genes and influences growth and cell division in *Corynebacterium glutamicum*

Kim Julia Kraxner, Tino Polen, Meike Baumgart*, and Michael Bott*

IBG-1: Biotechnology, Institute of Bio- und Geosciences, Forschungszentrum Jülich, 52425 Jülich, Germany

*Corresponding authors:
E-mail m.baumgart@fz-juelich.de; phone +49 2461 615515
E-mail m.bott@fz-juelich.de; phone +49 2461 613294

Content

Supplementary methods 3

Coulter counter measurements 3

Quantitative PCR........ 3

Genome resequencing 3

Supplementary results 4

Genome-resequencing of ATCC13032Δ*ftsR* uncovers amplification of the DNA region encompassing cg0828 – cg0840........ 4

Influence of FtsR on *ftsZ* promoter activity in strains with FtsR-independent *ftsZ* expression....... 4

Table S1. MALDI-TOF-MS analysis of the proteins enriched by DNA affinity chromatography with the *ftsZ* promoter region. 5

Table S2. Transcriptome comparison of the *C. glutamicum* strains ATCC13032Δ*ftsR* and ATCC13032 using DNA microarrays[a]. 6

Table S3. Amplification of cg0828-cg0840 in the ATCC13032Δ*ftsR* mutants. 8

Table S4. Bacterial strains and plasmids used in this study. 10

Table S5. Oligonucleotides used for DNA affinity chromatography, cloning, and EMSAs......... 12

Figure S1. 16

Figure S2. 17

Figure S3. 18

Figure S4. 19

Figure S5. 20

Figure S6. 21

Figure S7. 22

Figure S8. 23

Figure S10. 25

Figure S11. 26

Figure S12. 27

Figure S13. 28

Figure S14. 29

References 30

Supplementary methods

Coulter counter measurements

Bacterial size distribution was determined via the Coulter Principle using a MultiSizer 3 (Beckman Coulter, Krefeld, Germany) particle counter equipped with a 30 µm capillary. Briefly, for each measurement the bacterial cells at an $OD_{600\ of}$ ~0.1 were 20-fold diluted in CASYton assay buffer (Shärfe Systems, Reutlingen/Germany). Each sample (100 µl) was analyzed twice in the volumetric measurement mode. The data were visualized and extracted using the Beckman Coulter Multisizer 3 software package.

Quantitative PCR

To elucidate how many copies of the genes cg0834 and cg0840 were present in the genome of different *C. glutamicum* strains, quantitative PCR (qPCR) was performed. For this purpose, genomic DNA was isolated as following. Selected strains were incubated overnight in 5 mL BHI medium and harvested by centrifugation. The cells were washed in TE buffer (10 mM Tris-HCl, 1 mM Na_2EDTA, pH 7.6), centrifuged again and suspended in 1 mL TE buffer with 15 mg lysozyme. After three hours incubation at 37°C, 3 mL lysis buffer (10 mM Tris-HCl, 400 mM NaCl, 2 mM Na_2EDTA, pH 8.2), 220 µL 10% (w/v) SDS and 150 µL Proteinase K (20 µg/mL) were added to the suspension and mixed carefully, followed by an additional incubation for two hours at 60°C. 2 mL saturated saline solution was added and the samples were shaken vigorously until appearance of white precipitate, which was spun down by centrifugation for 30 minutes and 16.000 *g* at room temperature. The cleared supernatant was transferred into a fresh reaction tube and the DNA was precipitated by careful mixing with 2.5 volumes of ice cold ethanol absolute. The DNA was removed from the tube with a Pasteur pipette whose tip was bent before using a Bunsen burner, dipped into 70 % Ethanol in a fresh Eppendorf tube for washing, and air-dried for a few seconds. Subsequently, the DNA was solubilized in 200 µL TE buffer and incubated at 4°C overnight. For quality control, the isolated genomic DNA was analyzed by agarose gel electrophoresis. The DNA concentration was determined using a Colibri Microvolume Spectrophotometer (Berthold Detection Systems GmbH, Pforzheim, Germany) and adjusted to 50 ng/µL. For the qPCR experiment, PCR fragments for the generation of standard curves were amplified with chromosomal *C. glutamicum* DNA as template and the oligonucleotides given in table S5. The gene *recF* was used as reference gene, as it has proven to be well suitable in previous projects. The primers were designed with the Primer3plus online tool (1), using the standard settings for qPCR and an annealing temperature of 60°C. The amplified fragments were checked for purity by agarose gel electrophoresis. The standards were used in concentrations of 10 pg/µL to 100 ag/µL in a gradient cycle protocol to determine the optimal annealing temperature for the qPCR experiment. The setup of the cycling protocol was as following. 3 min pre-incubation at 95°C (step 1), 5 sec denaturation at 95°C (step 2), 25 sec elongation at a temperature-gradient from 55.1°C to 66.9°C (step 3), 40 times repetition of steps two and three, followed by a melting curve analysis (step 4) from 60°C to 95°C, with $\Delta T = 1$°C for every 6 seconds. Again, agarose gel electrophoresis of the PCR products was performed by agarose gel electrophoresis. Based on this experiment, an annealing temperature of 59°C was chosen for the qPCR experiment. The qPCR reaction was performed using the innuMIX qPCR MasterMix SyGreen (Analytik Jena, Jena, Germany) and the qTOWER 2.2 (Analytik Jena, Jena, Germany), and the protocol stated above. The reaction mix contained 10 µL master mix (2x), 1 µL primer 1 (10 µM), 1 µL primer 2 (10 µM), 2 µL template (50 ng/µL) and 6 µL H_2O. The data was analyzed using the program qPCRsoft 3.1 (Analytik Jena, Jena, Germany) and the ΔΔCt method.

Genome resequencing

For genome re-sequencing, *C. glutamicum* ATCC13032 and three independent clones of *C. glutamicum* ATCC13032Δ*ftsR* were cultivated overnight in 20 mL BHI medium and the DNA prepared (2). Genomic DNA was purified using the NucleoSpin® Microbial DNA Kit (MACHEREY-NAGEL GmbH & Co. KG, Düren, Germany). 4 µg were used for library preparation and indexing with the TruSeq® DNA PCR-Free Sample Preparation Kit (illumina Inc., San Diego, CA, USA). Quantifications of the resulting libraries were conducted using KAPA Library Quantification Kits (PEQLAB Biotechnologie GmbH, Erlangen, Germany) and were normalized for pooling. A MiSeq™ sequencing device (illumina Inc., San Diego, CA, USA) was used for paired-end sequencing with a read-length of two times 150 bases. Data analysis and base calling were accomplished with the illumina® instrument software and

stored as fastq output files. The sequencing data obtained were imported into CLC Genomics Workbench (Qiagen Aarhus A/S, Aarhus, Denmark) for trimming and base quality filtering. The output was mapped to accession BX927147 as the *C. glutamicum* ATCC13032 reference genome. The resulting mappings were used for the quality-based single nucleotide polymorphisms (SNPs) variant detection with CLC Genomics Workbench. The detected SNPs were manually inspected for relevance.

Supplementary results

Genome-resequencing of ATCC13032Δ*ftsR* uncovers amplification of the DNA region encompassing cg0828 – cg0840

The transcriptome comparison of ATCC13032Δ*ftsR* with ATCC13032 revealed 2.6- to 4.6-fold increased mRNA levels of the genomic region encompassing cg0830 – cg0838 in the Δ*ftsR* mutant (Table S3). Interestingly, two of the nine genes annotated in this region are oriented in the opposite direction than the other seven, but nevertheless also showed an increased mRNA level. Although this could be a consequence of a similar regulation of the nine genes, it might also result from an amplification event leading to an increased DNA copy number and therefore increased mRNA levels. We therefore sequenced the entire genome of three clones of strain ATCC13032Δ*ftsR*. For all three clones, this analysis indeed revealed an amplification event. As shown in Table S3, the sequence coverage of the DNA region from cg0828 to cg0840 was 5- to 7-fold (mean value 6.36 ± 0.57) higher than that of the residual genome in all three clones. When looking at the DNA microarray data, it becomes evident that the mRNA levels of cg0828, cg0829, cg0839, and cg0840 were also 2.5- to 3.4-fold increased in the Δ*ftsR* mutant, but since the p-value was above 0.05, they were not included in Table S2. The results of genome resequencing were confirmed by qPCR, which revealed a ≥5-fold increased DNA level for cg0834 and cg0840 compared to the reference gene *recF* (cg0005) in the ATCC13032Δ*ftsR* strain (Fig. S7). The mechanism of the DNA amplification is unknown and the exact genomic structure of the amplification cannot be deduced from the short sequencing reads. In four independent clones of MB001Δ*ftsR*, qPCR did not reveal an increased DNA level of cg0834 and cg0840 (Fig. S7), indicating that the amplification event only occurred in ATCC13032Δ*ftsR*, but not in the prophage-free strain MB001Δ*ftsR*. Since *ftsR* deletion mutants were constructed only once for each strain, firm conclusions on a functional correlation between the observed amplification event and the presence of the prophages cannot be drawn. However, the amplification could be responsible for the differences in the complementation studies between the wild type and the MB001 strain described in the main manuscript.

Influence of FtsR on *ftsZ* promoter activity in strains with FtsR-independent *ftsZ* expression.

The *ftsR* deletion in *C. glutamicum* MB001 led to a significant reduction of *ftsZ* promoter activity (Fig. 4). In order to confirm that the observed activation of the *ftsZ* promoter by FtsR is independent of the actual *ftsZ* expression, strains MB001::P_{gntK}-*ftsZ* and MB001Δ*ftsR*::P_{gntK}-*ftsZ* were transformed with the reporter plasmid pJC1-P_{ftsZ}-*venus* and either pEC-*ftsR* or pEC-XC99E as empty plasmid control (Fig. S13). With *ftsZ* being under control of P_{gntK} and addition of the same gluconate concentration to the medium, expression of the chromosomal *ftsZ* should be identical for all strains. In plasmid pEC-*ftsR* the *ftsR* gene is expressed under control of the IPTG-inducible but leaky *trc* promoter (Fig. S13). The growth conditions were chosen according to the previous experiment where the mixture of 0.01% (w/v) gluconate and 1.99% (w/v) glucose led to comparable growth of strains MB001::P_{gntK}-*ftsZ* and MB001Δ*ftsR*::P_{gntK}-*ftsZ*. The latter strain carrying pJC1-P_{ftsZ}-*venus* and the vector pEC-XC99E showed a significant growth defect in comparison to the other strains tested (Fig. S13A, red curve). This defect must be due to altered expression of genes besides *ftsZ* that are regulated directly or indirectly by FtsR. Accordingly, the growth defect was reversed in the presence of the *ftsR* expression plasmid (Fig. S13A, blue curve). The activity of the native *ftsZ* promoter on plasmid pJC1-P_{ftsZ}-*venus* was much lower in the strain lacking FtsR (Fig. S13B, red curve) and was strongly increased when *ftsR* was expressed via pEC-*ftsR* (Fig. S13B, blue curve). Growth and specific fluorescence was comparable for the two strains with chromosomal *ftsR* expression (Fig. S13, black and green curves). These results confirm transcriptional activation of *ftsZ* expression by FtsR and that the phenotype of *ftsR* deletion mutants is not solely caused by reduced *ftsZ* expression.

Table S1. MALDI-TOF-MS analysis of the proteins enriched by DNA affinity chromatography with the *ftsZ* promoter region.

Protein	Annotated function	Calculated mass (Da)	Mascot score[a]	Number of peptides matched	MS/MS[b] (ion score)	Sequence coverage
Cg1354	transcription termination factor Rho	83985	161	19		39%
Cg1525	DNA polymerase I	96769	248	30		44%
Cg1998	restriction endonuclease CglIIR	71053	181	23		43%
Cg2321	DNA polymerase III epsilon subunit	51383	68	10		24%
Cg0444	transcriptional regulator RamB	54117	202	23	1400.7 (35)<br>1559.7 (37)<br>1774.8 (31)	52%
Cg1997	type II restriction endonuclease CglIR	39915	104	12		47%
Cg1631	transcriptional regulator FtsR	27267	52	7	1103.5 (21)<br>1389.6 (23)<br>1566.8 (47)	30%
Cg3307	single-stranded DNA-binding protein Ssb	23287	46	4		30%

[a] The following parameters were used for the database search: database, in-house database of *C. glutamicum* ATCC13032 proteins based on the genome sequence; missed cleavages, 1; global modifications, carbamidomethyl (C); variable modifications: oxidation (M), mass tolerance: 80 ppm; MS/MS tolerance, 0.5 Da.

[b] Given are the masses of the peptides identified by MS/MS and the corresponding ion scores.

Table S2. Transcriptome comparison of the *C. glutamicum* strains ATCC13032Δ*ftsR* and ATCC13032 using DNA microarrays[a].

Locus tag	Gene name	Annotated function	mRNA ratio Δ*ftsR*/WT	p-value
cg0027		putative transcriptional regulator, MarR-family	2.39	0.034
cg0078		putative membrane protein	2.06	0.029
cg0256		putative protein, conserved	2.32	0.047
cg0274	*maoA*	putative oxidoreductase, possibly involved in oxidative stress response	2.51	0.048
cg0292	*tnp16a*	transposase	2.00	0.032
cg0661		hypothetical protein, conserved	2.33	0.040
cg0760	*prpB2*	2-methylisocitrate lyase, propionate catabolism	4.20	0.017
cg0762	*prpC2*	2-methylcitrate synthase, propionate catabolism	4.22	0.007
cg0797	*prpB1*	2-methylisocitrate lyase	2.03	0.018
cg0798	*prpC1*	2-methycitrate synthase	2.01	0.007
cg0824	*tnp5a*	transposase	2.44	0.003
cg0830		putative membrane protein	3.22	0.045
cg0831	*tusG*	ABC-type trehalose uptake system, permease	2.89	0.029
cg0832	*tusF*	ABC-type trehalose uptake system, permease	2.90	0.026
cg0833		putative membrane protein, involved in trehalose uptake	2.63	0.030
cg0834	*tusE*	ABC-type trehalose uptake system, binding protein	2.98	0.039
cg0836		hypothetical protein	3.30	0.027
cg0837		hypothetical protein	3.28	0.021
cg0838		putative helicase	4.61	0.026
cg1942		putative secreted protein CGP3 region	2.05	0.015
cg1969		hypothetical protein CGP3 region	2.03	0.026
cg1971		hypothetical protein CGP3 region	2.41	0.025
cg1995		hypothetical protein CGP3 region	2.23	0.042
cg2009		putative CLP-family ATP-binding protease, CGP3 region	2.22	0.047
cg2065		putative superfamily II DNA or RNA helicase, CGP3 region	2.83	0.021
cg2066		putative low-complexity protein	2.02	0.036
cg2183	*oppC*	ABC-type peptide transport system, permease component	8.82	0.015
cg2184	*oppD*	ABC-type peptide transport system, ATPase component	17.99	0.005
cg2461	*tnp4a*	transposase	2.23	0.031
cg2570	*dctP*	C4-dicarboxylate-binding protein, TRAP-family	2.10	0.018
cg2644	*clpP2*	ATP-dependent Clp protease proteolytic subunit	2.14	0.006
cg2915		hypothetical protein	2.61	0.021
cg3181		putative secreted protein	2.30	0.046
cg3266	*tnp5c*	transposase	2.13	0.001
cg0370		putative ATP-dependent RNA helicase, DEAD/DEAH box-family	0.50	0.015
cg0789	*amiA*	putative N-acyl-L-amino acid amidohydrolase	0.48	0.003
cg0812	*dtsR1 (accD1)*	acetyl/propionyl-CoA carboxylase, β chain, mycolic acid biosynthesis	0.47	0.025
cg0896		putative membrane protein	0.20	0.032
cg0980	*mepB*	putative secreted protein related to metalloendopeptidase	0.49	0.001
cg1037	*rpf2*	resuscitation promoting factor, secreted protein	0.42	0.015
cg1076	*glmU*	putative UDP-N-acetylglucosamine pyrophosphorylase	0.49	0.014

cg1290	*metE*	5-methyltetrahydropteroyltriglutamate-homocysteine methyltransferase, essential for methionine biosynthesis	0.39	0.036
cg1336		putative secreted protein	0.46	0.037
cg1370		hypothetical protein, conserved	0.39	0.026
cg1631	*ftsR*	transcriptional activator of *ftsZ*, MerR-family	0.02	0.004
cg2118	*fruR*	transcriptional regulator of sugar metabolism, presumably fructose responsive, DeoR-family	0.36	0.002
cg2366	*ftsZ*	cell division protein FtsZ	0.35	0.019
cg2477		hypothetical protein, conserved	0.24	0.007
cg2519		hypothetical protein, conserved	0.49	0.027
cg2853		putative protein fragment, conserved	0.45	0.006
cg3186	*cmt2*	trehalose corynomycolyl transferase	0.49	0.049
cg3335	*malE (mez)*	malic enzyme ($NADP^+$)	0.49	0.002

[a] Three biological replicates of the experiment were performed and genes are listed whose average mRNA ratio was increased or decreased at least 2-fold and whose p-value was ≤0.05.

Table S3. Amplification of cg0828-cg0840 in the ATCC13032Δ*ftsR* mutants.

Locus tag	Gene name	Annotated function	Coverage ATCC13032[a]	Coverage ATCC13032Δ*ftsR*[a]			mRNA Δ*ftsR*/WT[b]	p value
				clone 1	clone 2	clone 3		
cg0819		hypothetical protein	0.99	1.07	1.14	1.01	0.97	0.45
cg0820	*purE*	phosphoribosylamino-imidazole carboxylase, catalytic subunit	1.10	1.08	1.20	1.03	0.93	0.39
cg0821		conserved hypothetical protein	1.07	1.07	0.92	1.01	0.90	<0.01
cg0822		conserved hypothetical protein	1.02	1.03	0.94	0.99	1.38	0.02
cg0823	*ntaA*	nitrilotriacetate monooxygenase component A	1.06	0.89	0.99	0.93	0.85	0.32
cg0824		Tnp5a, transposase	0.98	1.50	1.57	1.50	2.44	<0.01
cg0825	*fabG*	3-ketoacyl-acyl-carrier-protein reductase	0.90	0.91	0.95	0.98	1.08	0.22
cg0826		putative membrane protein	1.10	1.32	1.34	1.21	0.91	0.33
cg0827		hypothetical protein	0.99	1.07	1.14	1.01	---	---
cg0828		putative dihydrofolate reductase	0.97	5.24	6.49	6.22	3.41	0.05
cg0829		putative lactoyl-glutathione lyase or related lyase, glyoxylase family	0.94	5.95	7.00	7.09	2.91	0.06
cg0830		putative membrane protein	0.99	5.6	6.42	6.48	3.22	0.05
cg0831	*tusG*	ABC-type trehalose uptake system, permease	1.03	5.42	5.93	6.23	2.89	0.03
cg0832	*tusF*	ABC-type trehalose uptake system, permease	0.96	5.46	5.68	6.18	2.90	0.02
cg0833		membrane protein,	1.01	6.18	6.74	7.34	2.63	0.03
cg0834	*tusE*	ABC-type trehalose uptake system, binding protein	1.10	5.90	6.35	7.02	2.98	0.04
cg0835	*tusK*	ABC-type trehalose uptake system, ATPase	1.03	5.51	6.28	6.72	2.27	0.06
cg0836		hypothetical protein	0.98	5.72	6.40	6.74	3.30	0.03
cg0837		hypothetical protein	1.03	6.12	6.28	6.73	3.28	0.02
cg0838		ATP-dependent helicase	1.05	6.40	7.10	7.28	4.61	0.03
cg0839		hypothetical protein	1.12	6.46	7.04	7.33	2.45	0.16
cg0840		conserved hypothetical protein	1.04	5.67	6.79	6.64	2.45	0.19
cg0841		conserved hypothetical protein	0.98	1.02	0.98	0.93	1.36	0.05

cg0842		putative DNA helicase	1.02	1.01	0.98	1.01	1.11	0.21
cg0843		putative helicase	0.98	0.95	0.99	0.95	1.10	0.21
cg0844		putative type II restriction enzyme, methylase subunit	1.06	1.03	1.00	0.96	1.57	0.05
cg0845		putative superfamily II DNA/RNA helicase, SNF2-family	0.97	0.94	1.03	0.89	0.61	0.06
cg0847		conserved transcriptional regulator	0.82	0.97	0.93	0.86	0.64	<0.01
cg0848	*wbbL*	putative rhamnosyl transferase	0.85	0.92	0.95	0.84	0.53	0.02
cg0849	*rmlA2*	GDP-mannose pyrophosphorylase, mannose-1-phosphate guanylyltransferase	0.98	1.02	1.02	0.96	0.61	0.14
cg0850	*whcD*	transcription factor, *whmD* homolog, not involved in oxidative stress	1.09	1.00	1.05	1.2	0.68	0.01

[a] Sequence coverage obtained from genome re-sequencing of ATCC13032 and three clones of strain ATCC13032Δ*ftsR*. The mean coverage of the entire genome of ATCC13032 was set as 1. The genes showing a 5- to 7-fold increased coverage in the Δ*ftsR* clones are underlined.

[b] mRNA ratios with the corresponding p-values obtained by DNA microarray analysis

Table S4. Bacterial strains and plasmids used in this study.

Strain or plasmid	Characteristics	Source or reference
**Bacterial strains**		
E. coli		
DH5α	F$^-$ Φ80*dlac*Δ(*lacZ*)M15 Δ(*lacZYA-argF*) U169 *endA1 recA1 hsdR17* (r_K^- , m_K^+) *deoR thi-1 phoA supE44* λ$^-$ *gyrA96 relA1*; strain used for cloning procedures	Hanahan (3)
C. glutamicum		
ATCC13032	Biotin-auxotrophic wild type	Kinoshita, Udaka (4)
ATCC13032Δ*ftsR*	ATCC13032 with in-frame deletion of *ftsR* (cg1631).	This work
ATCC13032Δ*ramB*	ATCC13032 with in-frame deletion of *ramB* (cg0444)	Gerstmeir, Cramer (5)
ATCC13032::*ftsZ-venus*	ATCC13032 with a chromosomal insertion of pK18*mob-ftsZ-venus* at the *ftsZ* locus. This strain has two chromosomal copies of *ftsZ* under control of its native promoter, one with and one without the fusion to *venus.*	This work
ATCC13032Δ*ftsR*::*ftsZ-venus*	ATCC13032Δ*ftsR* with a chromosomal insertion of pK18*mob-ftsZ-venus* at the *ftsZ* locus. This strain has two chromosomal copies of *ftsZ* under control of its native promoter, one with and one without the fusion to *venus.*	This work
MB001	ATCC13032 with in-frame deletion of prophages CGP1 (cg1507-cg1524), CGP2 (cg1746-cg1752), and CGP3 (cg1890-cg2071)	Baumgart, Unthan (6)
MB001Δ*ftsR*	MB001 with in-frame deletion of *ftsR* (cg1631)	This work
MB001::P_{gntK}-*ftsZ*	MB001 with a chromosomal promoter exchange of the native *ftsZ*-promoter against the gluconate inducible promoter of *gntK* (cg2732)	This work
MB001Δ*ftsR*::P_{gntK}-*ftsZ*	MB001::P_{gntK}-*ftsZ* with in-frame deletion of *ftsR*	This work
***C. diphtheriae* ATCC27010**	Standard strain used for analysis of *C. diphtheriae*; genomic DNA used as PCR template	DSM 44123
***M. tuberculosis* H37Rv**	Standard strain for analysis of *M. tuberculosis*; genomic DNA used as PCR template	ATCC25618
**Plasmids**		
pK19*mobsacB*	Kan^R; plasmid for allelic exchange in *C. glutamicum*; (pK18 *ori*$V_{E.c.}$, *sacB*, *lacZ*α)	Schäfer, Tauch (7)
pK19*mobsacB*-Δ*ftsR*	Kan^R; pK19*mobsacB* derivative for in-frame deletion of *ftsR*; harbouring a PCR product covering the upstream and downstream regions of *ftsR*	This work
pK19*mobsacB*-P_{gntK}-*ftsZ*	Kan^R; pK19*mobsacB* derivative for construction of MB001::P_{gntK}-*ftsZ* harbouring a PCR fragment containing 691 bp of the upstream region of *ftsZ* (cg2366) covering its promoter followed by a terminator sequence, the *gntK* (cg2732) promoter and 523 bp of the *ftsZ* coding region	This work

Strain or plasmid	Characteristics	Source or reference
pK18*mob*	Kan^R; plasmid for insertion of a DNA fragment into the chromosome of *C. glutamicum*; (pK18 *ori*V$_{E.c.}$, *lacZ*α)	(7)
pK18*mob-ftsZ-venus*	Kan^R; pK18*mob* derivative for chromosomal insertion of the coding sequence for a fusion protein of FtsZ and the fluorescent protein Venus under control of the native *ftsZ* promoter	This work
pK19-P2732-*lcpA*	Kan^R; pK19*mobsacB* derivative used as PCR template during construction of the promoter exchange plasmid pK19*mobsacB*-P$_{gntK}$-*ftsZ*	Baumgart, Schubert (8)
pAN6	Kan^R; *C. glutamicum*/*E. coli* shuttle vector for regulated gene expression using P$_{tac}$ (P$_{tac}$ *lacI*^{q} pBL1 *oriV*$_{Cg}$ pUC18 *oriV*$_{Ec}$)	(9)
pAN6-*ftsR*	Kan^R; pAN6 derivative for expression of *ftsR* (252 amino acids) under control of P$_{tac}$	This work
pAN6-*ftsR*-Strep	Kan^R; pAN6 derivative for expression of *ftsR* under control of P$_{tac}$ with a C-terminal StrepTag-II	This work
pAN6-*ftsR*-short	Kan^R; pAN6 derivative encoding an N-terminally shorted FtsR protein (224 amino acids) under control of P$_{tac}$	This work
pAN6-CDC7B_1201	Kan^R; pAN6 derivative for expression of *C. diphtheriae* CDC7B_1201 (*ftsR* homolog) under control of P$_{tac}$	This work
pAN6-rv1828	Kan^R; pAN6-derivative for expression of *M. tuberculosis* rv1828 (*ftsR* homolog) under control of P$_{tac}$	This work
pEC-XC99E	Cm^R; *C. glutamicum*/*E. coli* shuttle vector for regulated gene expression using P$_{trc}$, (*lacI*^{q}, *oriV*$_{E.c}$, *per*, *repA* (pGA1)$_{C.g.}$)	Kirchner and Tauch (10)
pEC- *ftsR*	Cm^R; pEC-XC99E derivative for expression of *ftsR* under control of P$_{trc}$	This work
pJC1-*venus*-term	Kan^R; pJC1 derivative carrying the Venus coding sequence and additional terminators (pCG1 *ori*$_{Cg}$, pACYC177 *ori*$_{Ec}$)	Baumgart, Luder (11)
pJC1-P$_{ftsZ}$-*venus*	Kan^R; pJC1-*venus*-term derivative with the *ftsZ* promoter controlling expression of *venus* for promoter activity studies	This work

Table S5. Oligonucleotides used for DNA affinity chromatography, cloning, and EMSAs.

Oligonucleotide	Sequence (5’ → 3’) and properties[a]	Commentary[b]
**DNA affinity purification with the *ftsZ* promoter region**		
AP_pftsZ_fw	CATTAGCTCACCCTCAATGG	
AP_pftsZ_rv_bio	*GAGGAGTCGTCGATGTGGAGACC*GAGGCCTTCTTCAATCATGC	overlap (in italics) homologous to the biotinylated oligonucleotide used as reverse primer in a second PCR to create a biotinylated fragment
biotin_oligo	5'BIO-GAGGAGTCGTCGATGTGGAGACC	biotinylated oligonucleotide homologous to the overlap of the primer AP_pftsZ_rv_bio for attachment of a biotin tag to the resulting fragments; biotinylated molecules bind with high affinity to the Streptavidin-coated magnetic beads used for DNA affinity purification.
**Construction of pK19*mobsacB*-derivatives and pK18*mob*-derivatives**		
M13-fw	CGCCAGGGTTTTCCCAGTCAC	for sequencing of pK19*mobsacB*-Δ*ftsR* and pK18*mob-ftsZ-venus*
M13-rv	AGCGGATAACAATTTCACACAGGA	
**Deletion plasmid pK19*mobsacB*-Δ*ftsR***		
D_ftsR_1_fw	GATCGGATCCTCCGCACTCAACATCTAGAC	PCR product contains BamHI site for cloning in BamHI-cut pK19*mobsacB*
D_ftsR_2_rv	TGTTTAAGTTTAGTGGATGGGGATGTTGCTGCTACCTGCTGTGAATTAAA	
D_ftsR_3_fw	CCCATCCACTAAACTTAAACATAGAAAAAATGAGTTTTGTTGAACTT	PCR product contains HindIII site for cloning in HindIII-cut pK19*mobsacB*
D_ftsR_4_rv	GATCAAGCTTCGTCGCCTGAAGCAGATTCC	
map_DftsR_fw	TCCGCACTCAACATCTAGAC	for verification of chromosomal *ftsR* deletion
map_DftsR_rv	CAGGTAAAGCCATCTGGTTC	
**Promoter exchange plasmid pK19*mobsacB*-P_{gntK}-*ftsZ***		
ftsZ_upstream_fw	**CAGGTCGACTCTAGAGGATC**GAAGTGCTTCCTGCGGTTAT	for amplification of a fragment upstream of the *ftsZ* coding region with homologies to the pK19*mobsacB* backbone and to the terminator sequence
ftsZ_upstream_rv	**CACTACCATCGGCGCTAC**TGTCGATGTCTCGCCTTTCG	
Term_fw	GTAGCGCCGATGGTAGTG	for amplification of a fragment harbouring the terminator and the P_{gntK} coding sequence; template: pK19-P2732-cg0847 (8)
Pcg2732-rv	GTCTTATCCTTTCTTTGGTGGCG	
PgntK_ftsZ_fw	**CACCAAAGAAAGGATAAGAC**ATGACCTCACCGAACAACTA	for amplification of a fragment of the first 523 bp of the *ftsZ* (cg2366) coding region including start codon with homologies to the P_{gntK} sequence and the pK19*mobsacB* backbone
ftsZ_start_rv	**AAAACGACGGCCAGTGAATT**GTCGTTTGGAATAACGATGA	
map_term-PgntK_insertion_fw	TGAGTGCGGAACCAGCTTCG	for verification of insertion of the terminator-P_{gntK}-fragment between P_{ftsZ} and the *ftsZ* coding region on the chromosome
map_term-PgntK_insertion_rv	ACCATCACCGTGGAGCTGAC	

**Plasmid pK18*mob-ftsZ-venus* for chromosomal insertion of *ftsZ-venus***		
pK18_IRG-fw-V2	**CCTGCAGGTCGACTCTAGAG**GTTTACGCAGCACAAGACCCC	for amplification of a fragment covering about 450 bp of the intergenic region between cg1121 and cg1122
IGR-rv	TCCTCCATAATTAGAGAGCGTAAGGCCC	
PftsZ-fw	**CGCTCTCTAATTATGGAGGA**TGATGGTGACCATGTCATTGACACCG	for amplification of the promoter and the coding region of *ftsZ*
FtsZ-rv	**TCCTCGCCCTTGCTCACCAT**CTGGAGGAAGCTGGGTACATCCAG	
venus-fw	ATGGTGAGCAAGGGCGAGGAG	For amplification of the *venus* gene encoding the fluorescent protein Venus
pK18_venus-rv-V2	**CAGCTATGACCATGATTACG**TTACTTGTACAGCTCGTCCATGCC	
FtsZ-Seq1	**CATTAGCTCACCCTCAATGGTG**	For sequencing of pK18*mob-ftsZ-venus*
FtsZ-Seq2	**GAACCTGTCCATCATGGAAGC**	
int-reg-fw	AGCACCTTCGGCAAGAAGTA	Test of integration strains for integration into the intergenic region of cg1121-cg1122.
int-reg-rv	CATCGAAGGTGTCGCAAAC	
ftsQ-rv	AGCAATAACCGCAGGAAGCAC	
M13-fw	CGCCAGGGTTTTCCCAGTCAC	
ftsQ-fw	ACAAGGCAGGACTAGCGTGAAC	Test of integration strains for integration into the chromosomal *ftsZ*-region.
ftsZ-downstream-rv	TCGTGAAGACCTTGCGGAC	
pK18-IGR-fw	CTTGGTTCGAATATGCAGTTCGG	
eYFP-int_rv	CGACCAGGATGGGCACCAC	
**Construction of pAN6 derivatives**		
pAN6_check_fw	CATCGGAAGCTGTGGTATGG	for sequencing of pAN6-derivatives
pAN6_check_rv	CCTGGCAGTTCCCTACTCTC	
**pAN6-FtsR, pAN6-FtsR-short & pAN6-FtsR-Strep**		
pAN6_ftsR_fw	**CTGCAGAAGGAGATATACATATGA**GTGCACTCCGTAAAAC	PCR product with overlaps for cloning of *ftsR* in the NdeI-/EcoRI-cut pAN6 plasmid; with fw-primer, the *ftsR* start codon GTG is changed to ATG
pAN6_ftsR_short_fw	**CTGCAGAAGGAGATATACATATG**TCAATTGGTGTGGTACT	
pAN6_ftsR_rv	**AAAACGACGGCCAGTGAATT**TCAGTATCCAAGCTGCTCGC	
pAN6_ftsR_Strep_rv	**CTGTGGGTGGGACCAGCTAGT**GTATCCAAGCTGCTCGCGA	with pAN6_ftsR_fw amplification of a PCR product without stop codon with overlaps for in-frame cloning of *ftsR* upstream of a StrepTag-II coding region for expression of FtsR-Strep in the NdeI-/NheI-cut pAN6 vector
**pAN6-CDC7B_1201**		
CDC7B_1201_pAN6_fw	**GCCTGCAGAAGGAGATATACAT**ATGAGTGCACTTCCGCAACG	PCR product with overlaps for cloning of CDC7B_1201 gene (*ftsR* homolog) in the NdeI-/EcoRI-cut pAN6 vector; template: *C. diphtheriae* ATCC27010 genomic DNA
CDC7B_1201_pAN6_rv	**AAAACGACGGCCAGTGAATT**TTAACGGTTCAGCTCATCGCGC	
**pAN6-rv1828**		
rv1828_pAN6_fw	**GCCTGCAGAAGGAGATATACAT**GTGAGCGCACCCGATAGCC	PCR product with overlaps for cloning of Rv1828 gene (*ftsR* homolog) in the NdeI-/EcoRI-cut pAN6 vector; template: *M. tuberculosis* H37Rv genomic DNA
rv1828_pAN6_rv	**AAAACGACGGCCAGTGAATT**TCAGCGGTGAAGAACGTCGCGAAC	

**Construction of pEC-*ftsR***		
ftsR_BamHI_fw	GATCGGATCCGCCTGCAGAAGGAGATATAC	PCR product contains BamHI and XbaI sites for cloning in pEC-XC99E vector cut with the same enzymes; template: pAN6-*ftsR*, amplified fragment harbours RBS from pAN6
ftsR_XbaI_rv	GATCTCTAGATCAGTATCCAAGCTGCTCGC	
pEC-check_fw	TAATCATCGGCTCGTATAATGTGTG	for sequencing of pEC-derivatives
pEC-check_rv	GCTTCTGCGTTCTGATTTAATCTG	
**Construction of pJC1-P_{ftsZ}-*venus***		
pJC1_PftsZ_fw	**AGCGACGCCGCAGGGGGAT**CTTCCTGCGGTTATTGCTGTA	amplification of the *ftsZ* promoter region equipped with overlaps homologous to the pJC1-*venus*-term vector backbone cut with SpeI and to the *venus* coding region
PftsZ_venus_rv	**CTCCTCGCCCTTGCTCAC**CATTGTCGATGTCTCGCCTTTCG	
venus_fw	ATGGTGAGCAAGGGCGAGGAG	PCR product with overlap for cloning into BamHI-cut pJC1-*venus*-term; template: pJC1-*venus*-term
venus_pJC1_rv	**AAAACGACGGCCAGTACTAG**TTACTTGTACAGCTCGTCCATGC	
pJC1_check_fw	TGAAGACCGTCAACCAAAGG	for sequencing of pJC1-*venus*-term derivatives
pJC1_check_rv	TACGGCGTTTCACTTCTGAG	
**30 bp oligonucleotides for electrophoretic mobility shift assays**		
ftsZ_oligo	CGCTACCCTCAACCTTTACTTTAGGGTTGT	
ftsZ_oligo_compl	ACAACCCTAAAGTAAAGGTTGAGGGTAGCG	
cg1081_oligo	GAAGCCACATGACATATGTCATGAAAATTA	
cg1081_oligo_compl	TAATTTTCATGACATATGTCATGTGGCTTC	
Competition-EMSA		
Cy3-ftsZ_prom_rv	Cy3* TCTTGGCGAGGTAGTTGTTC	5'-Cy3-label
ftsZ_prom_rv	TCTTGGCGAGGTAGTTGTTC	
ftsZ_prom250_fw	AGTTCCGGTTCACCCGTTTC	
ftsZ_prom500_fw	AGTGCTTCCTGCGGTTATTG	
ftsZ_prom250up_rv	CTACCCAGCCACTTTAGCGG	

[a] Restriction sites are underlined. Bold letters represent the overlapping sequences needed for Gibson assembly.
[b] If not stated otherwise, *C. glutamicum* ATCC13032 genomic DNA was used as template.

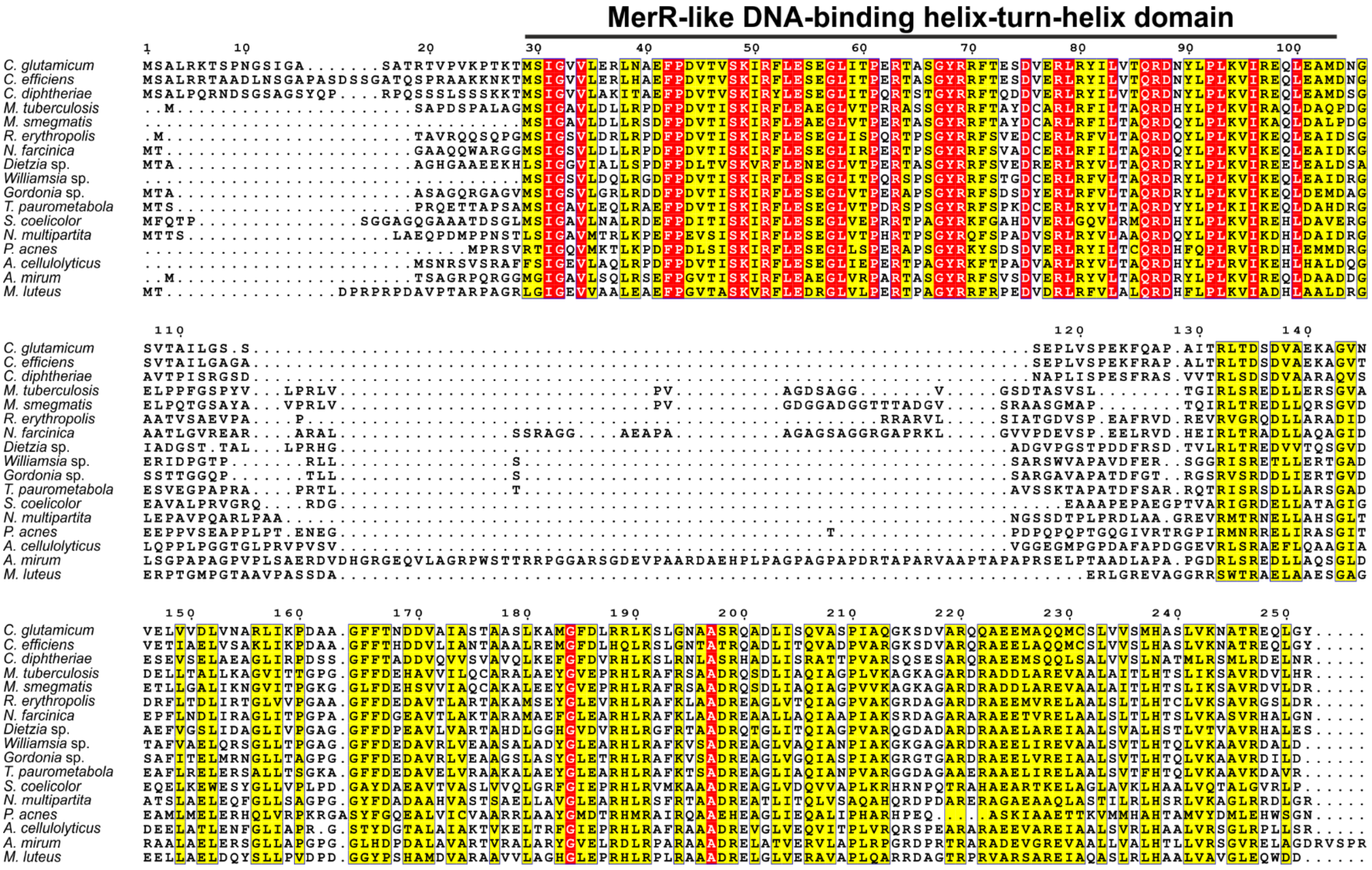
MerR-like DNA-binding helix-turn-helix domain
C. glutamicum
C. efficiens
C. diphtheriae
M. tuberculosis
M. smegmatis
R. erythropolis
N. farcinica
Dietzia sp.
Williamsia sp.
Gordonia sp.
T. paurometabola
S. coelicolor
N. multipartita
P. acnes
A. cellulolyticus
A. mirum
M. luteus

Figure S1. Alignment of *C. glutamicum* FtsR and homologous proteins of other *Actinobacteria*. The alignment was prepared using Clustal Omega (12) and edited using ESPript 3.0 (13). Residues shown in yellow are at least 70% identical and residues indicated in red are fully conserved. The following proteins were aligned: *Corynebacterium glutamicum* FtsR (Cg1631); *Corynebacterium efficiens* CE1574; *Cornyebacteirum diphtheriae* DIP_1205; *Mycobacterium tuberculosis* Rv1828; *Mycobacterium smegmatis* MSMEG_3646; *Rhodococcus ruber* CS378_RS03820; *Nocardia farcinica* NFA_24870; *Dietzia* sp. H483_RS33310; *Williamsia* sp. ASG12_08425; *Gordonia effusa* GOEFS_105_00530; *Tsukamurella paurometabola* Tpau_2338; *Streptomyces coelicolor* SCO1383; *Nakamurella multipartita* NAMU_RS19840; *Propionibacterium acnes* PPA_RS05480; *Acidothermus cellulolyticus* ACEL_RS06295; *Actinosynnema mirum* AMIR_RS26540; *Micrococcus luteus* CRM77_RS01150.

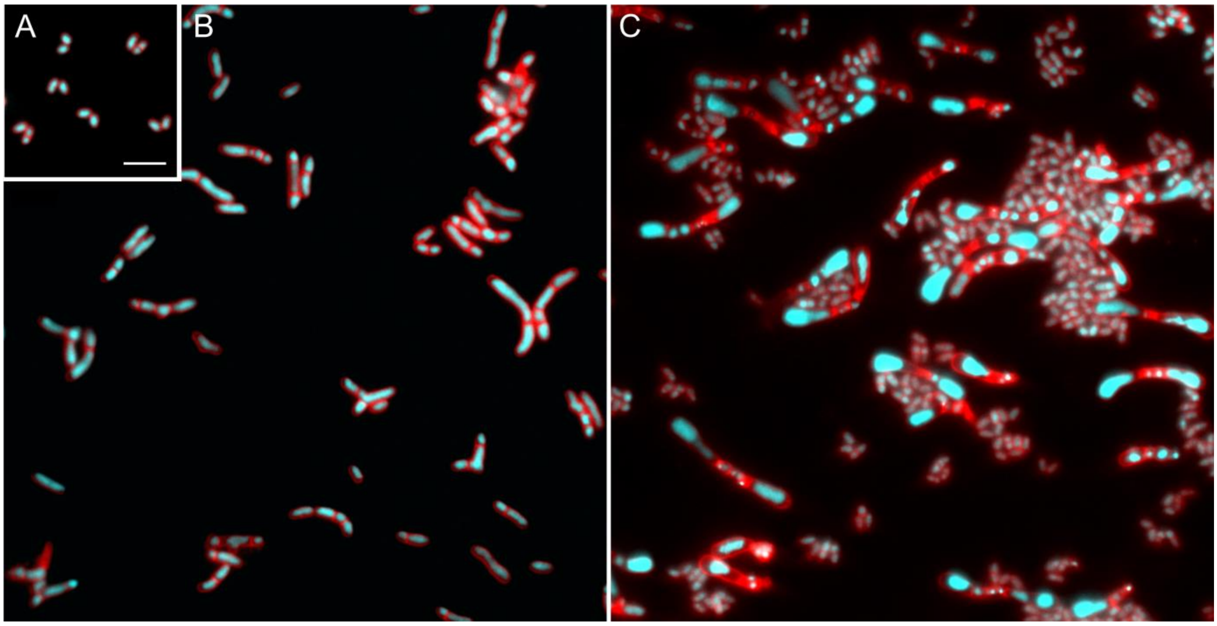

Figure S2. Morphology of *C. glutamicum* ATCC13032 (A) ATCC13032Δ*ftsR* (B) and ATCC13032 pAN6-*ftsR* (C). The cells were first cultivated in BHI medium followed by two consecutive cultivations in CGXII minimal medium with 2% (w/v) glucose as carbon source. For the plasmid-based overexpression (C), kanamycin (25 µg/mL) and IPTG (100 µM) were added. Fluorescence microscopy of stationary phase cells was performed. DNA was stained with Hoechst 33342 (cyan) and membranes with Nile red (red) as described in the Methods section. The scale bar is 5 µm.

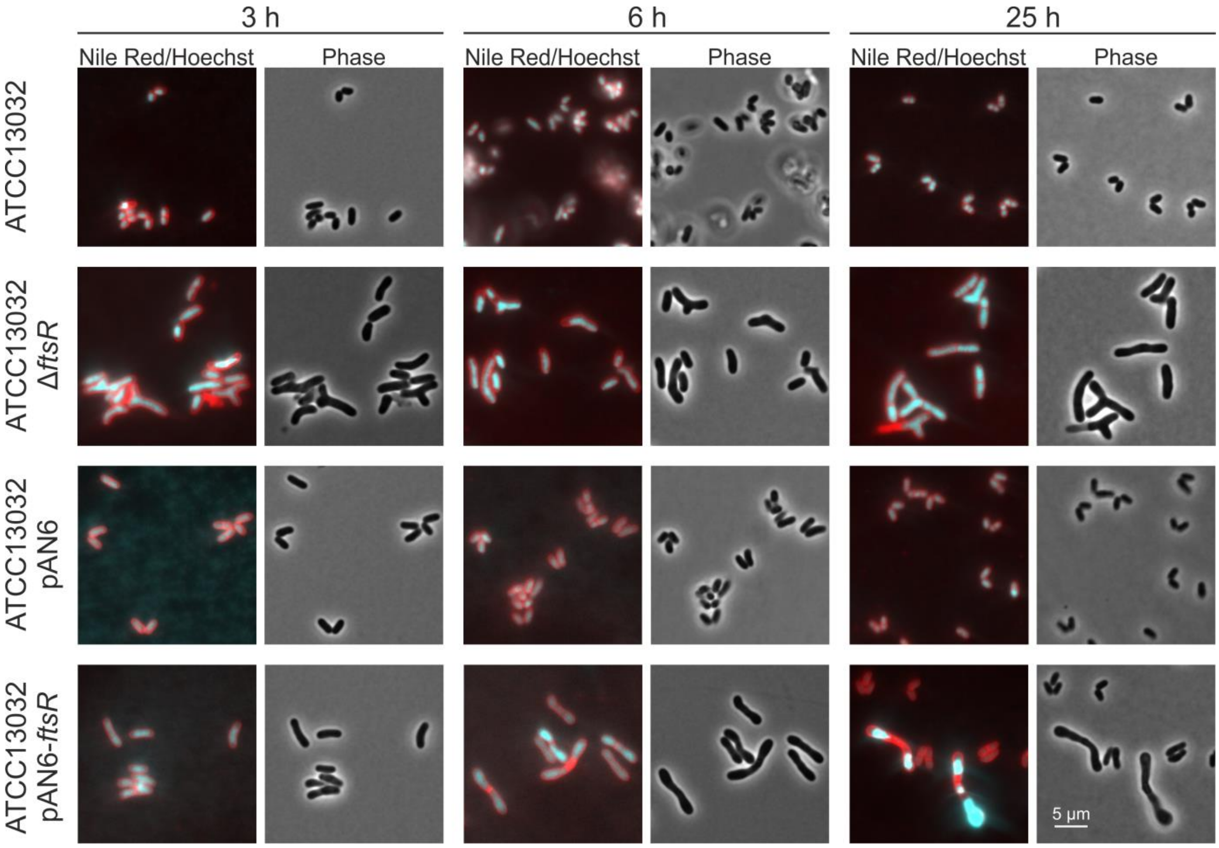

Figure S3. Morphology of *C. glutamicum* ATCC13032, ATCC13032Δ*ftsR*, ATCC13032 pAN6, and ATCC13032 pAN6-*ftsR* in different growth phases. The cells were first cultivated in BHI medium followed by two consecutive cultivations in CGXII minimal medium with 2% (w/v) glucose as carbon source. For the strains carrying plasmids, kanamycin (25 µg/mL) and IPTG (100 µM) were added. Fluorescence microscopy was performed with staining of DNA with Hoechst 33342 (cyan) and membranes with Nile red (red) as described in the Methods section.

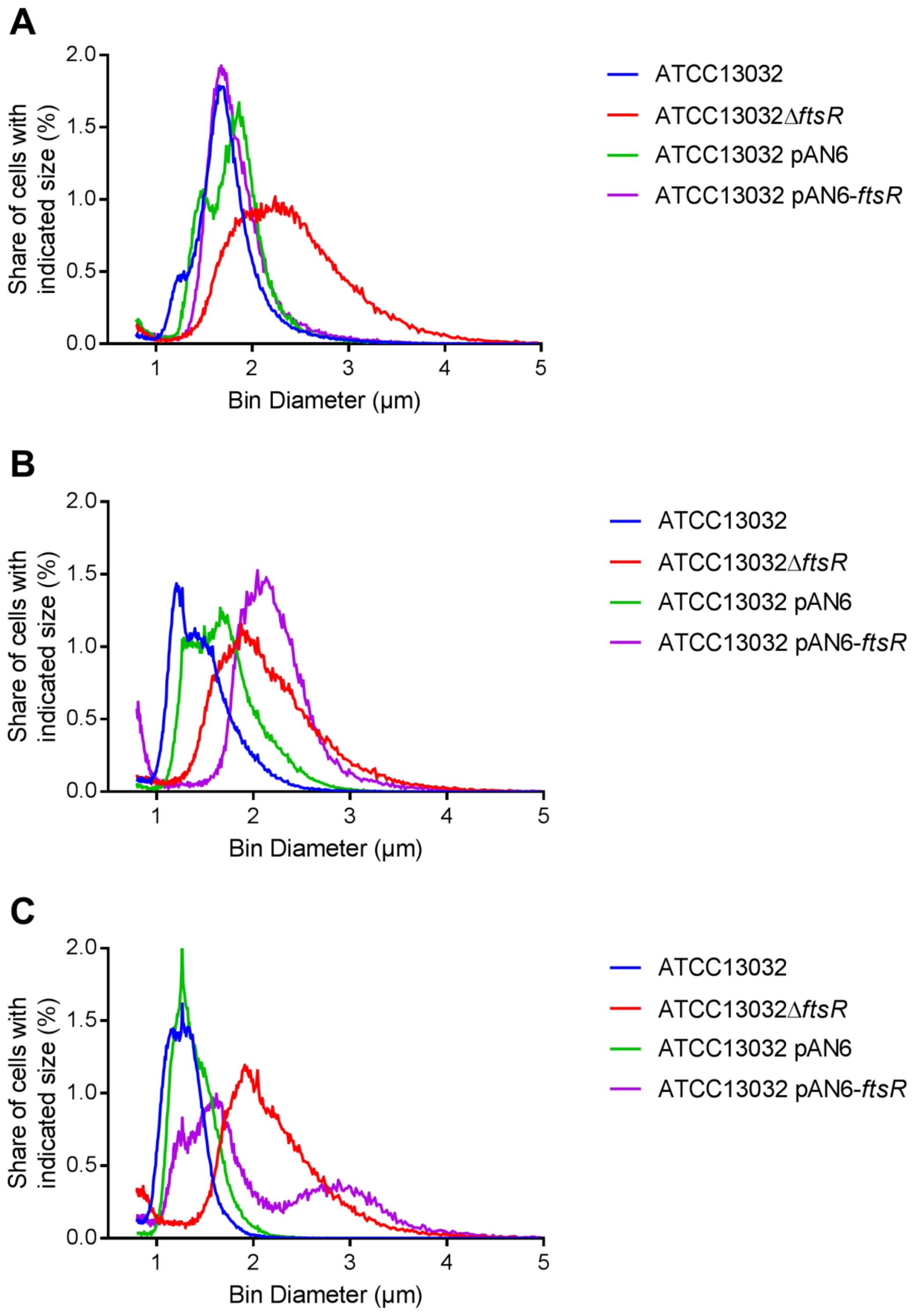

Figure S4. Coulter Counter measurements of the indicated strains in the exponential phase at 3 h (A) and 6 h (B) and in the stationary phase at 24 h (C). The cells were diluted to an OD_{600} of ~0.1 and analyzed in the volumetric measurement mode. The cells were assigned to bins according to their size. The bin diameter is proportional to the cell size. Plotted are mean values of two technical replicates.

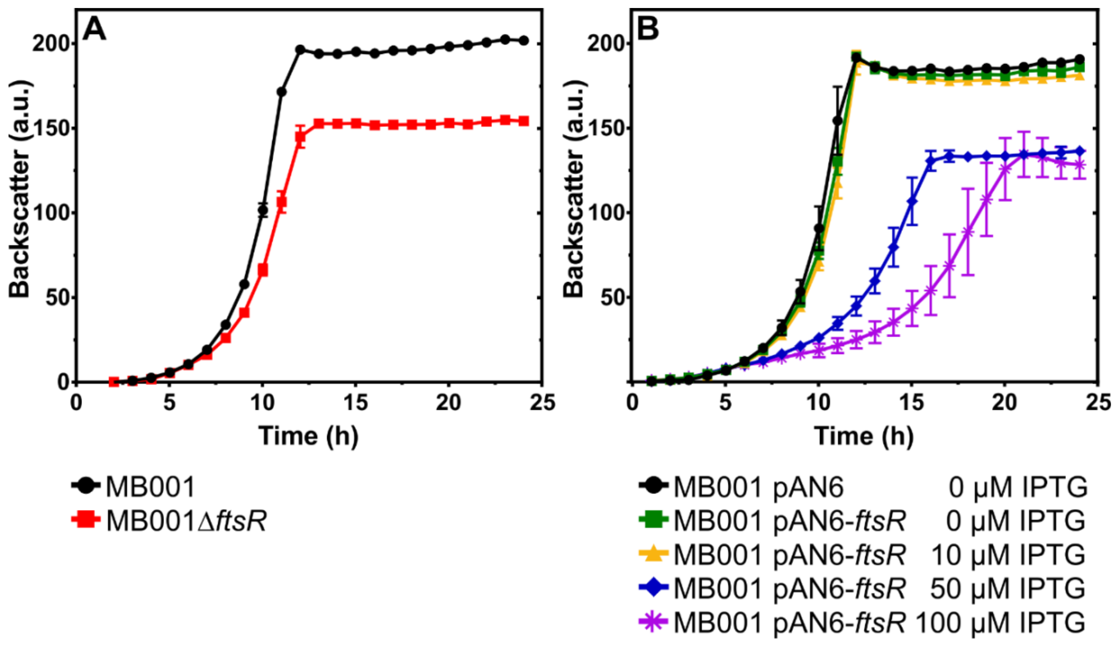

Figure S5. Growth behavior of *C. glutamicum* MB001 with deleted (A) or (over)expressed (B) FtsR. The cells were first cultivated in BHI medium followed by two consecutive cultivations in CGXII minimal medium with 2% (w/v) glucose as carbon source. For the plasmid-based (over)expression, kanamycin (25 µg/mL) and IPTG was added as indicated. Averages and standard deviations of three biological replicates of the second CGXII culture are presented.

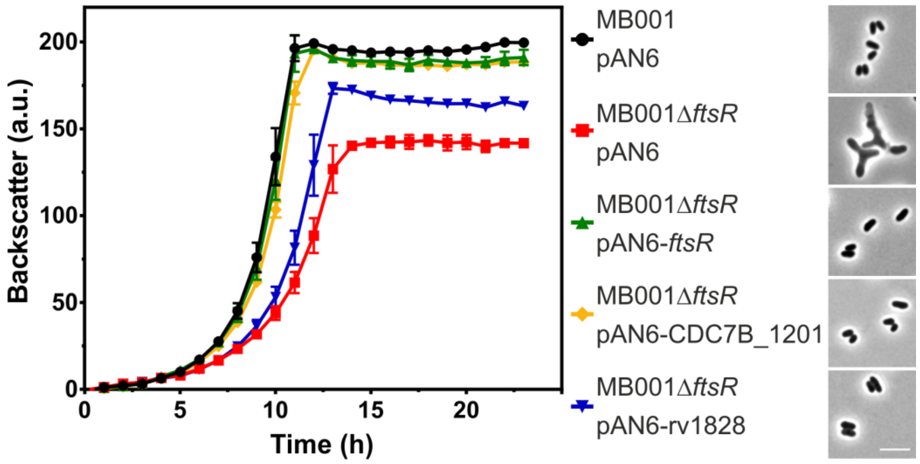

Figure S6. Complementation of the growth defect (left panel) and the morphological phenotype (right panel) of the MB001Δ*ftsR* mutant by plasmid-encoded FtsR and homologous proteins of related pathogenic species. The cells were first cultivated in BHI medium with kanamycin (25 µg/mL) followed by two consecutive cultivations in CGXII minimal medium with kanamycin (25 µg/mL) and 2% (w/v) glucose as carbon source. MB001Δ*ftsR* was transformed with pAN6 encoding *ftsR*, CDC7B_1201, or rv1828 under control of the leaky *tac* promoter. IPTG was only added for expression of rv1828, where 100 µM were required to obtain optimal complementation. Averages and standard deviations of three biological replicates of the second CGXII culture are presented. Microscopy was performed with stationary phase cells. The scale bar represents 5 µm.

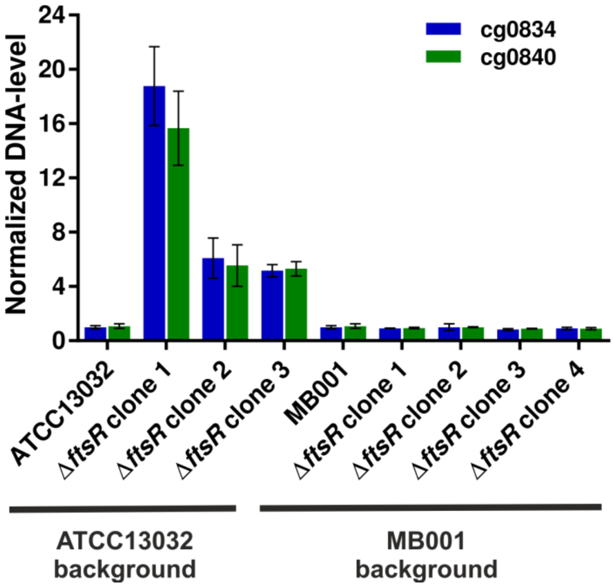

Figure S7. Normalized DNA levels of cg0834 and cg0840 in different *ftsR* deletion strains and the corresponding reference strains. Chromosomal DNA of ATCC13032, MB001, and several Δ*ftsR* clones of the two strains was isolated and the relative copy numbers were determined. The DNA level of *recF* was used as reference and was set to 1. Illustrated are the normalized DNA levels of the genes cg0834 and cg0840 determined by qPCR and calculated using the ΔΔCt quantification with the program qPCRsoft 3.1.

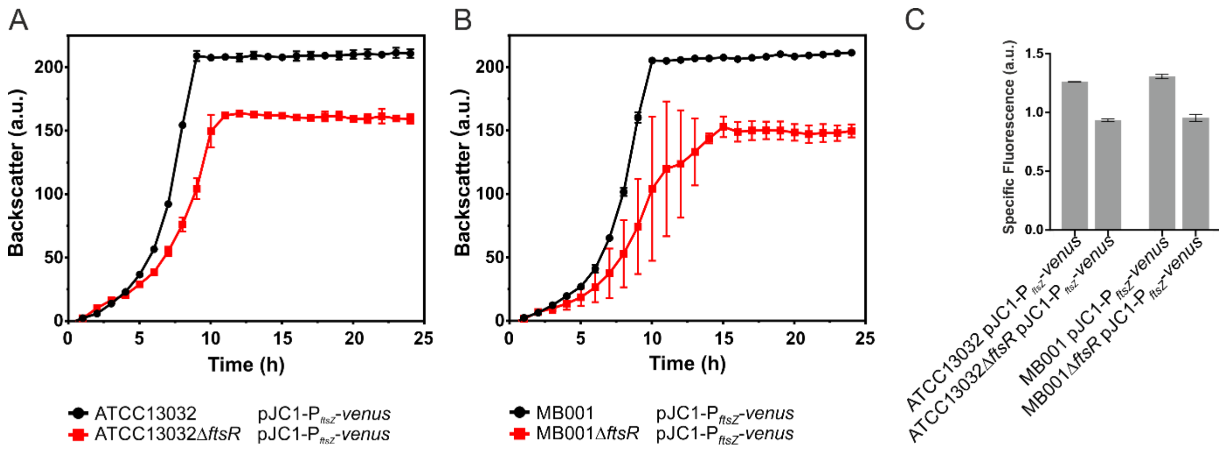

Figure S8. Influence of the *ftsR* deletion on the expression of the venus reporter gene under control of the *ftsZ* promoter. The indicated strains were cultivated in CGXII medium with 1% (w/v) glucose and 25 µg/ml kanamycin in a BioLector microcultivation system with automated measurement of cell density as backscatter at 620 nm and fluorescence (excitation at 510 nm, emission at 532 nm). Specific fluorescence represents the ratio of fluorescence and backscatter. (A) Results for the ATCC13032 background. (B) Results for the MB001 background. (C) Comparison of the specific fluorescence of all strains after 24 h. Averages and standard deviations of three biological replicates are shown.

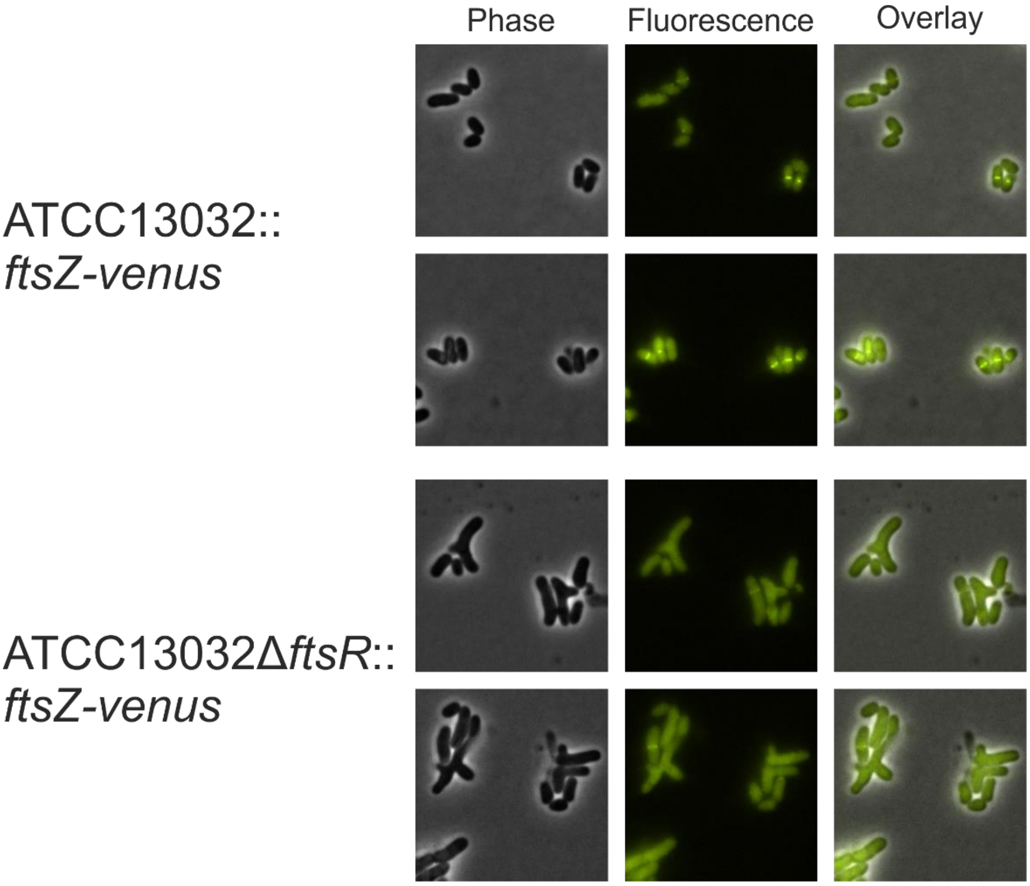

Figure S9. Fluorescence microscopy of the *C. glutamicum* strains ATCC13032::*ftsZ-venus* and ATCC13032Δ*ftsR*::*ftsZ-venus* carrying a second chromosomal copy of *ftsZ* fused in frame to the coding sequence of the fluorescent protein Venus. The cells were first cultivated in BHI + 2 % (w/v) glucose and afterwards transferred to CGXII + 2 % (w/v) glucose, both with 25µg/mL kanamycin. Samples for microscopy were taken after about 6 h cultivation of the main culture, which is approximately in the middle of the exponential growth phase. Two representative pictures for each culture are shown. The experiment was performed with two biological replicates each.

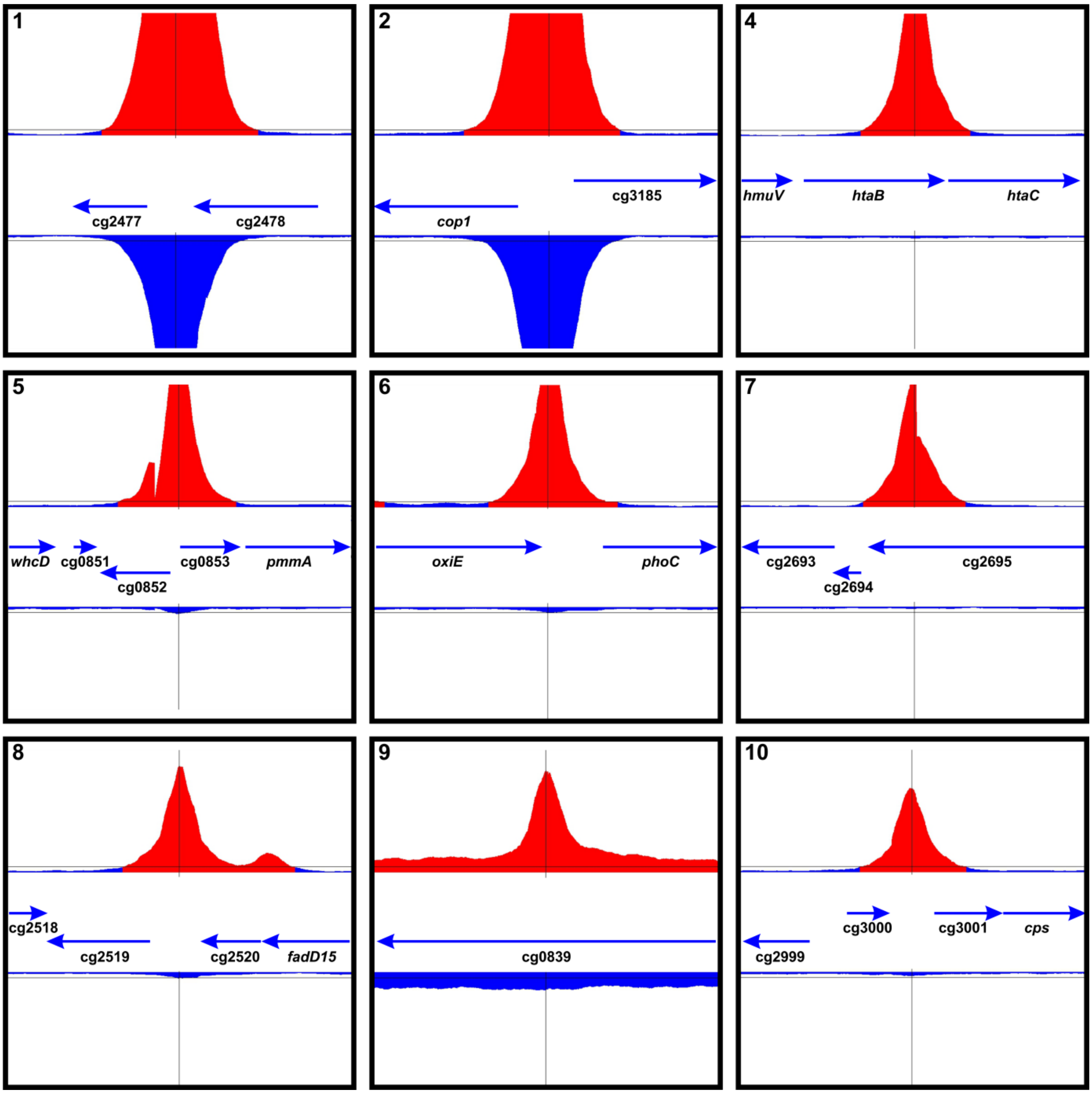

Figure S10. DNA regions showing the highest coverage (red peaks) in the ChAP-Seq experiment with FtsR-Strep (strain *C. glutamicum* Δ*ftsR* pAN6-*ftsR*-Strep). As negative control, the DNA enriched by StrepTactin affinity chromatography from strain *C. glutamicum* Δ*ftsR* pAN6 was used (blue background). The red peak in the *ftsZ* promoter region (between cg2365 and cg2366), which had the 3rd highest coverage, is depicted in Fig. 5. Due to the high peaks in the negative control of sample peaks 1 and 2, an independent ChAP-Seq experiment was performed, which confirmed binding of FtsR between cg2477 and cg2478 and between *cop1* and cg3185 (see Table 1) without the high background peaks in the negative control.

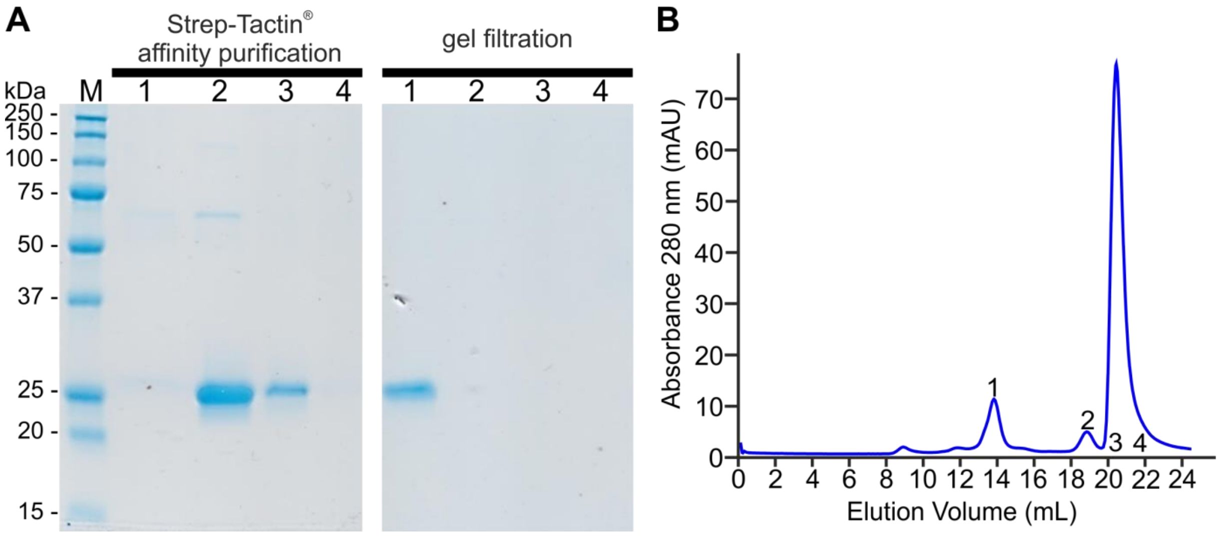

Figure S11. Purification of FtsR-Strep. (A) SDS-PAGE of purified FtsR-Strep. On the left site, the elution fractions 1 – 4 of the Strep-Tactin® affinity purification are shown. The second elution fraction was subjected to gel filtration for further purification (see Materials and Methods for details) and the analyzed fractions marked in the elution profile of the gel filtration (B) are shown on the right site of the gel. Gel filtration was performed using a Superdex™ 200 Increase 10/300 GL column attached to an Äkta Pure25 system at a flow rate of 0.6 mL/minute (GE Healthcare Bio-Sciences AB, Uppsala, Sweden) and a buffer composed of 50 mM Tris-HCl, 250 mM NaCl, pH 7.5. The molecular mass of FtsR was estimated by comparison with standard proteins of known molecular mass and elution volume, which were cytochrome *c* (12.4 kDa, 18.82 mL), carbonic anhydrase (29 kDa, 16.15 mL), albumin (66.0 kDa, 13.73 mL), alcohol dehydrogenase (150 kDa, 12.53 mL), and β-amylase (200 kDa, 11.58 mL). FtsR-Strep eluted at 13.70 mL.

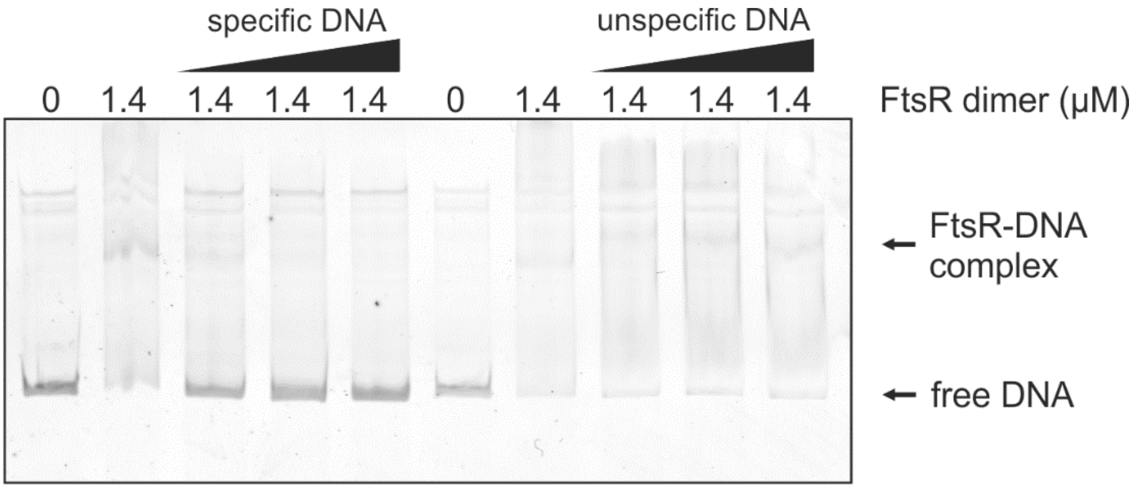

Figure S12. EMSA competition experiment with FtsR and the *ftsZ* promoter region. 2 nM Cy3-labeled *ftsZ* promoter DNA (271 bp) was incubated with 0 or 1.4 µM FtsR dimer and increasing concentrations (0.47 µM, 0.94 µM, 1.86 µM) of either unlabeled specific competitor DNA (same DNA fragment as above) or unlabeled unspecific competitor DNA (260 bp DNA fragment further upstream in the *ftsZ* promoter).

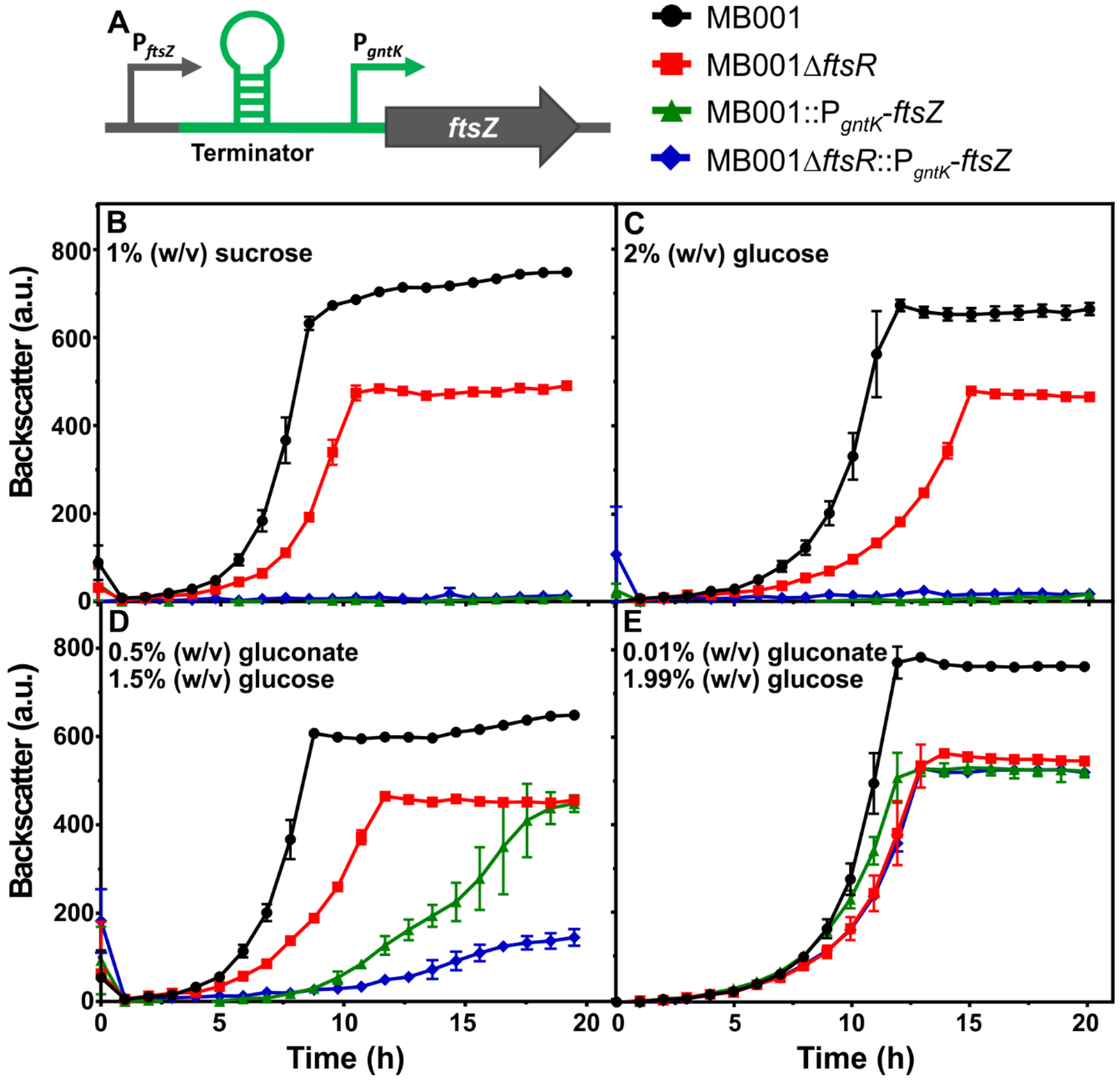

Figure S13. Promoter exchange of *ftsZ* and growth of the resulting strains. (A) Strains with FtsR-independent *ftsZ* expression were constructed using a DNA fragment with a terminator sequence and the gluconate-inducible *gntK* promoter, which was inserted between the native *ftsZ* promoter and the *ftsZ* start codon in the chromosomes of MB001 and the MB001Δ*ftsR* mutant. (B-D) The growth of the resulting promoter exchange strains was tested in comparison to the strains with the native *ftsZ* promoter using either sucrose (B), glucose (C), or glucose plus gluconate (C) as carbon source(s). (E) Cultivation with a gluconate/glucose ratio which enables a similar growth behavior of MB001::P_{gntK}-*ftsZ* and MB001Δ*ftsR*::P_{gntK}-*ftsZ*. Cells were pre-cultivated either in BHI medium (B and C) or in BHI medium supplemented with 0.1% (w/v) gluconate for P_{gntK} induction when gluconate was also used as carbon source in the main culture (D and E), followed by two consecutive cultivations in CGXII minimal medium supplemented with the indicated carbon sources. Averages and standard deviations from three biological replicates are presented.

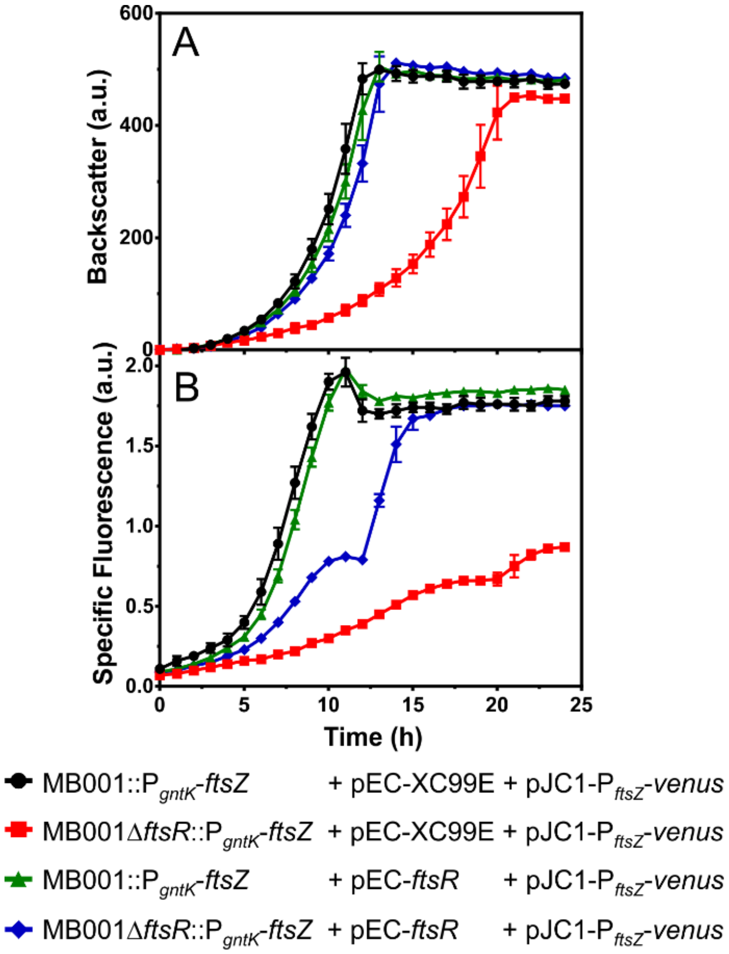

Figure S14. Influence of *ftsR* expression on (A) growth and (B) plasmid-based *ftsZ* promoter activity in the *ftsZ* promoter exchange strains MB001::P$_{gntK}$-*ftsZ* and MB001Δ*ftsR*::P$_{gntK}$-*ftsZ*. The two strains were transformed with plasmid pJC1-P$_{ftsZ}$-*venus* for monitoring *ftsZ* promoter activity and either with the *ftsR* expression plasmid pEC-*ftsR* or the vector pEC-XC99E. Cells were cultivated first in BHI complex medium supplemented with 0.1% (w/v) gluconate followed by a second pre-culture and the main culture in CGXII minimal medium containing 0.01% (w/v) gluconate and 1.99% (w/v) glucose as carbon source. Kanamycin (25 µg/mL) and chloramphenicol (10 µg/mL) were added to all cultures. Averages and standard deviations from three biological replicates are shown.

References

1. Untergasser A, Cutcutache I, Koressaar T, Ye J, Faircloth BC, Remm M, et al. Primer3—new capabilities and interfaces. Nucleic Acids Res. 2012;40(15):e115-e.
2. Pfeifer E, Gätgens C, Polen T, Frunzke J. Adaptive laboratory evolution of *Corynebacterium glutamicum* towards higher growth rates on glucose minimal medium. Sci Rep. 2017;7(1):16780.
3. Hanahan D. Studies on transformation of *Escherichia coli* with plasmids. J Mol Biol. 1983;166(4):557-80.
4. Kinoshita S, Udaka S, Shimono M. Studies on amino acid fermentation. Part I. Production of L-glutamic acid by various microorganisms. J Gen Appl Microbiol. 1957;3:193-205.
5. Gerstmeir R, Cramer A, Dangel P, Schaffer S, Eikmanns BJ. RamB, a novel transcriptional regulator of genes involved in acetate metabolism of *Corynebacterium glutamicum*. J Bacteriol. 2004;186(9):2798-809.
6. Baumgart M, Unthan S, Rückert C, Sivalingam J, Grünberger A, Kalinowski J, et al. Construction of a prophage-free variant of *Corynebacterium glutamicum* ATCC 13032 for use as a platform strain for basic research and industrial biotechnology. Appl Environ Microbiol. 2013;79(19):6006-15.
7. Schäfer A, Tauch A, Jäger W, Kalinowski J, Thierbach G, Pühler A. Small mobilizable multi-purpose cloning vectors derived from the *Escherichia coli* plasmids pK18 and pK19: Selection of defined deletions in the chromosome of *Corynebacterium glutamicum*. Gene. 1994;145(1):69-73.
8. Baumgart M, Schubert K, Bramkamp M, Frunzke J. Impact of LytR-CpsA-Psr proteins on cell wall biosynthesis in *Corynebacterium glutamicum*. J Bacteriol. 2016;198(22):3045-59.
9. Frunzke J, Engels V, Hasenbein S, Gätgens C, Bott M. Co-ordinated regulation of gluconate catabolism and glucose uptake in *Corynebacterium glutamicum* by two functionally equivalent transcriptional regulators, GntR1 and GntR2. Mol Microbiol. 2008;67(2):305-22.
10. Kirchner O, Tauch A. Tools for genetic engineering in the amino acid-producing bacterium *Corynebacterium glutamicum*. J Biotechnol. 2003;104(1-3):287-99.
11. Baumgart M, Luder K, Grover S, Gätgens C, Besra GS, Frunzke J. IpsA, a novel LacI-type regulator, is required for inositol-derived lipid formation in *Corynebacteria* and *Mycobacteria*. BMC Biol. 2013;11:122.
12. Sievers F, Wilm A, Dineen D, Gibson TJ, Karplus K, Li W, et al. Fast, scalable generation of high-quality protein multiple sequence alignments using Clustal Omega. Mol Syst Biol. 2011;7:539.
13. Robert X, Gouet P. Deciphering key features in protein structures with the new ENDscript server. Nucleic Acids Res. 2014;42:W320-4.
